# Identification of hub gene associated with colorectal cancer: Integrating Mendelian randomization, transcriptome analysis and experimental verification

**DOI:** 10.1371/journal.pgen.1011788

**Published:** 2025-07-29

**Authors:** Yu Zhang, Xu Han, Yichun Yang, Jiayan Ren, Zixi Zhang, Yanmin Zhang, Qi Su, Dake Chu

**Affiliations:** 1 School of Pharmacy, Health Science Center, Xi’an Jiaotong University, Xi’an, Shaanxi, P.R. China; 2 Department of Dermatology, The First Affiliated Hospital of Xi’an Jiaotong University, Xi’an, Shaanxi, P. R. China; 3 Department of Gastroenterology, The First Affiliated Hospital of Xi’an Jiaotong University, Xi’an, Shaanxi, P. R. China; Kaiser Permanente Research Bank, UNITED STATES OF AMERICA

## Abstract

**Background:**

Colorectal cancer (CRC) is a prevalent malignancy with significant mortality rates globally. Understanding the genetic and molecular mechanisms underlying CRC development is crucial for improving therapeutic strategies.

**Method:**

In this study, we utilized cis-eQTL summary data to identify genes potentially causally associated with CRC. The expression levels of candidate genes in tumor and normal tissues were compared using the GEPIA2 database. The correlations between FUT8 expression and cellular functions, tumor mutation burden, immune checkpoint genes, and immune infiltration were analyzed. Molecular docking was performed to identify potential drugs targeting FUT8, and the effects of the selected drug on cell proliferation were evaluated using the MTT assay. Additionally, the cellular thermal shift assay (CETSA) was employed to assess the interaction between the drug and the target protein.

**Results:**

We identified 19 genes with eQTLs potentially associated with CRC, among which six eQTLs were associated with increased CRC risk, including FUT8. FUT8 was significantly overexpressed in CRC tumor tissues and correlated with various cellular functions such as stemness, invasion, EMT, and metastasis. Higher FUT8 expression was associated with higher tumor mutation burden and significant correlations with multiple immune checkpoint genes. Molecular docking identified VE-822 as a promising drug candidate targeting FUT8, which demonstrated inhibitory effects on CRC cell proliferation. The CETSA results indicated that VE ‒ 822 could bind to FUT8 and improve its thermal stability.

**Conclusion:**

FUT8 is a crucial gene that causes colon cancer and is linked to tumour immunity. VE-822 is a promising candidate for treating CRC by targeting FUT8.

## Author summary

This study utilized cis-eQTL data to identify genes associated with colorectal cancer (CRC). The FUT8 gene, identified via SMR analysis, was found to be overexpressed in tumor tissues and correlated with cellular functions and immune checkpoint genes, highlighting its potential as a therapeutic target. Furthermore, molecular docking identified VE-822 as a promising drug candidate targeting FUT8. It demonstrated inhibitory effects on CRC cell proliferation and could bind to FUT8 and affect its expression. Our findings highlight the importance of the FUT8 gene in the development of colorectal cancer and suggest the potential of VE-822 as a treatment for colon cancer.

## 1. Introduction

Colorectal cancer (CRC) is a common intra-abdominal malignant tumor, accounting for approximately 10% of the annual diagnosed cancer cases and cancer-related deaths worldwide, making it the second leading cause of cancer-related mortality [1]. In recent years, the incidence of CRC has been showing a youth-oriented tendency. It is estimated that within the next 10 years, approximately 10% to 12% of CRC cases will be diagnosed in individuals under the age of 50 [2,3]. Therefore, it is imperative to gain a deeper understanding of the pathomechanisms of CRC and to explore new therapeutic approaches.

Quantitative trait locus (QTLs) analysis is a powerful tool for understanding the mechanisms that shape complex diseases by linking genetic variants identified in Genome-wide association studies (GWAS) to candidate susceptibility genes. The study of expression quantitative trait locus (eQTLs) has become an effective means of providing a genetic perspective to unravel the underlying biological mechanisms of complex diseases such as cancers, neurodegenerative disorders, and autoimmune disorders [4–6]. By studying these genetic variations, it is possible to uncover the underlying mechanisms of disease progression and identify new drug targets that may have been previously unknown or undervalued.

This study employed Summary-data-based Mendelian Randomization (SMR) method to identify genes that may have causal effects with CRC. eQTLs from eQTLGen were used as exposures. Further transcriptomic analysis of the key gene also validated its prognostic relevance in CRC and its possible influence on tumor immunity. This suggests that FUT8 could serve as a potential biomarker and therapeutic target for CRC. In this context, we also explored potential therapeutic strategies targeting these genes to provide new ideas for CRC treatment.

## 2. Methods

### 2.1. Multi-omics mendelian randomization

The SMR software tool was initially developed to implement the SMR & HEIDI method, which effectively integrates data from GWAS and QTLs studies. The eQTLs were obtained from the publicly available eQTLGen Consortium database (https://eqtlgen.org/), which contains 16,987 genes and 31,684 cis-eQTLs [7]. Genetic variants were extracted using a threshold of **P* *< 5 × 10^-8^. Linkage Disequilibrium (LD) Correction: Genetic variants in close proximity on the genome often exhibit LD, meaning they tend to be inherited together. This can lead to inflated false-positive rates in association studies if not properly addressed. By applying a clustering procedure with a strict threshold of R² < 0.001 within a truncation range of 10 Mb, we effectively removed the effects of LD and identified independent SNPs. This ensures that the genetic variants included in our analysis are not confounded by LD, thereby improving the reliability of our results.

The data for CRC GWAS were obtained from FinnGen R9 (https://r9.risteys.finngen.fi/endpoints/C3_COLON_ADENO) (S5 Table). The dataset included 3084 cases and 287137 controls. The screened eQTLs data were used as exposure factors, and the CRC GWAS dataset was used as outcome factors. The significance thresholds of the eQTLs analysis were subsequently adjusted for multiple testing using the FDR method. The positive eQTLs were visualized using the R package forestplot_3.1.3, which generated corresponding forest plots. In conclusion, we have identified genetic variations with a *P*_FDR _< 0.05 and *P*_HDIEI _> 0.05 as eQTLs causally related to CRC. The SMR analysis was consistent with the STROBE-MR guideline (S1 STROBE MR Checklist).

### 2.2. Gene expression analysis

To assess the expression differences of risk genes identified by SMR analysis between normal and colorectal cancer tissues, we utilized the GEPIA2 database, which compares tumor tissues from The Cancer Genome Atlas (TCGA) with normal tissues from the Genotype-Tissue Expression (GTEx) project. The Human Protein Atlas database (HPA, https://www.proteinatlas.org) provides comprehensive data on the distribution and expression of various proteins across 48 normal human tissues, 20 tumor tissues, and 47 cell lines using immunohistochemistry [8]. By integrating the immunohistochemical analysis of colorectal cancer patient tissues and data on colorectal cancer disease staging from the HPA database, we selected FUT8 as the target gene for subsequent studies.

### 2.3. Cellular function

The expression of FUT8 at the cellular level was analyzed using the Tumor Immune Single-cell Hub 2 (TISCH2) [9]. The parameters employed in the analysis included FUT8 (gene), major lineages (cell type annotation), and CRC (cancer type). The CancerSEA database (http://biocc.hrbmu.edu.cn/CancerSEA/) was employed to analyze the average correlation between the expression of FUT8 in different cancer single-cell datasets and the activity of each functional state. This analysis aimed to investigate the influence of FUT8 on 14 different functions [10].

### 2.4. Tumor mutation burden

High tumor mutation burden (TMB) has become a hallmark of immune therapy response [11,12]. TMB was studied in the CRC cohort of the TCGA database. It is known that cancer patients with a higher number of genetic mutations tend to generate more neoantigens, which increases the likelihood of recognition by immune cells. The aim was to assess the impact of increasing TMB on the occurrence of CRC. The number or frequency of mutations within the FUT8 gene region was analyzed using the R package maftools version 2.16.0. If there is a higher correlation between the target gene and tumor mutation burden, it is more likely that FUT8 will have a role in immune therapy.

### 2.5. Immune checkpoint correlation

Immune checkpoints refer to a series of protein molecules expressed in immune cells that regulate the degree of immune activation. The clinical advances achieved with drugs that block immune checkpoints have brought immunotherapy into the mainstream of oncology [13]. This study utilized the limma 3.54.2 package and reshape2 1.4.4 package to perform immune checkpoint analysis. The co-expression relationship between FUT8 and 79 immune checkpoint genes was investigated to assess their correlation. The significance threshold for the correlation analysis was set at a p-value of less than 0.001 (p < 0.001) and a correlation coefficient (Pearson’s r) of greater than 0.4 (r > 0.4). These thresholds were chosen to ensure that only strong and statistically significant correlations were considered. The corrplot 0.92 package was used for visualization purposes.

### 2.6. Immune infiltration analysis

The gene expression matrix of CRC patients downloaded from TCGA was used to analyze immune infiltration. To assess the extent of immune cell infiltration, the deconvolution algorithm CIBERSORT was employed. While this is a commonly used method in bioinformatics research [14], it is important to note that relying solely on a single algorithm may introduce certain limitations. To ensure objectivity, we used the R package ‘immunedeconv’ to perform immunoinfiltration analysis with eight different algorithms: Estimate, timer, ABIS, ConsensusTME, xCell, EPIC, quanTIseq, and the classical algorithm CIBERSORT. These algorithms were used to characterize immune cell populations in colon samples. Finally, we analyzed the immunoinfiltration differences between high and low FUT8 expression using multiple algorithms.

### 2.7. Molecular docking

To identify potential small-molecule drugs that can bind to FUT8, we screened our small-molecule drug library. Molecular docking simulations were performed to examine the interaction between FUT8 and potential drugs, with the drugs serving as ligands and FUT8 as the protein receptor. The 3D structures of the candidate compounds were sourced from PubChem, while the 3D structure of FUT8 was obtained from the Protein Data Bank (PDB) (https://www.rcsb.org/structure/2DE0). The results were evaluated using cb-Dock2. The optimal ligand-receptor binding configuration was selected based on a thorough analysis of the binding conformation and affinity. Intermolecular forces were carefully analyzed, and the binding energy of the optimal configuration was accurately calculated. The results of the molecular docking validation were confidently visualized using PyMol software.

### 2.8. Cell proliferation effected by VE-822

The MTT assay was employed to ascertain the inhibitory ability of VE-822 on the proliferation of SW480 cells. SW480 cells in a state of normal growth were seeded uniformly in 96-well plates at a density of 1x10^5^ cells/mL. Five parallel wells were set up in each group, and after the cells had adhered to the bottom of the wells after eight hours, different concentrations of VE-822 were added and returned to the cell incubator. Following 24, 48, and 72 hours of continuous proliferation, 10μL of MTT solution was added to each well to achieve a final concentration of 0.5mg/mL. The cells were then incubated for six hours at 37°C in a 5% CO_2_ incubator. The upper liquid layer was carefully aspirated and 150μL of DMSO was added to dissolve the crystals at the bottom of the well. The 96-well plate was shaken on an oscillator for 10 min to dissolve all crystals, and then the absorbance values of each group were examined at 490 nm.

### 2.9. Expression of FUT8 in NCM460 and SW480

The SW480 and NCM460 cells were cultured separately. Based on the key role of FUT8 protein as a CRC risk score, the difference in FUT8 protein expression in SW480 and NCM460 cells was detected using Western blot.

### 2.10. Cellular thermal shift assay

The SW480 cells in logarithmic phase were seeded in a medium dish. Once the cell density reached 80%, the cells were treated with VE-822 for 4 hours. The cells were then collected and washed twice with PBS. They were re-suspended in PBS and divided into five aliquots. These were incubated in a water bath at 40, 45, 50, 55, or 60°C for 4 min. Subsequently, the cells were lysed by repeated freeze-thawing three times in liquid nitrogen. The lysate was then centrifuged at 17,000g for 20 min at 4°C. The supernatant was collected and loading buffer was added. The protein was denatured in a metal bath at 100°C for 10 min and stored at -20°C. Western blot was used to detect the expression of FUT8 protein in different groups.

### 2.11. Statistical analysis

The statistical analysis methods and plotting tools used in this study were performed in RStudio, Rtools and R. The configuration environment and versions of R packages for each analysis are provided in S2 File. Data are expressed as the mean ± SEM. Statistical comparisons were performed using Student’s t-test. A significance level of **P* *< 0.05 was used throughout the study. All analyses were conducted using GraphPad Prism software (version 9.5.1).

## 3. Results

### 3.1. Identification of the hub gene in eQTLs

**Fig 1:**
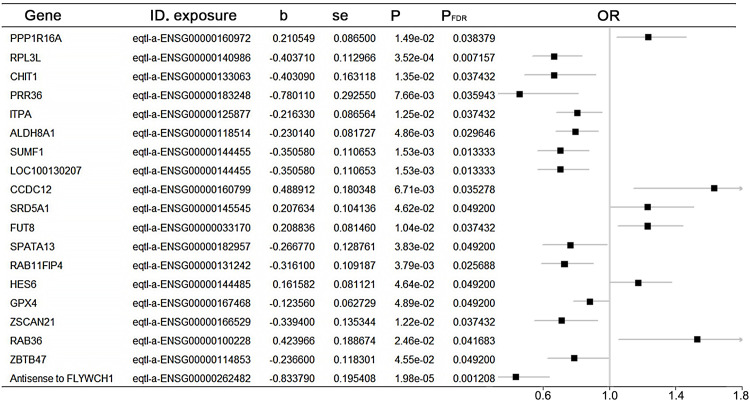
Positive eQTLs potentially causally associated with CRC.

**Fig 2:**
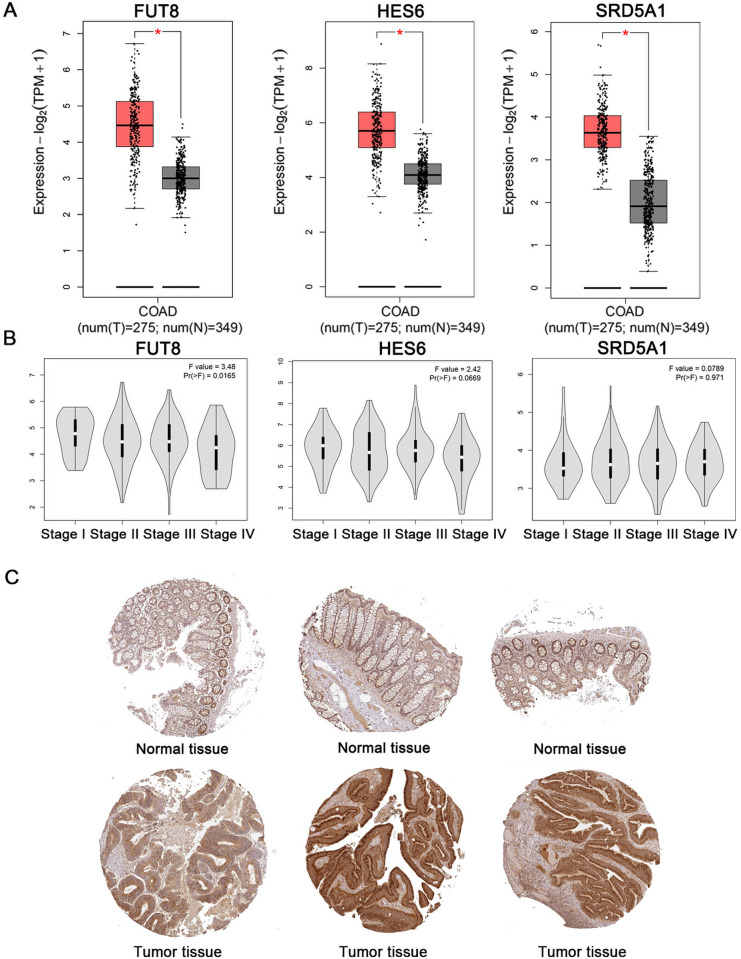
FUT8 expression levels and localization. A: The expression difference of related genes between CRC tumor tissue and normal tissue. B: Correlation between gene expression and clinical stage. C: Immunohistochemical observation of FUT8 in CRC tissues (n = 3). Immunohistochemical results of FUT8 protein were obtained from HPA database.

Using cis-eQTL summary data, 19 genes were identified as having eQTLs potentially causally associated with colorectal cancer (CRC). Among these, six eQTLs were found to be associated with an increased risk of CRC: PPP1R16A, CCDC12, SRD5A1, FUT8, HES6, and RAB36. Additionally, 13 eQTLs were identified as protective factors against CRC (Fig 1, S1 Table). Using the GEPIA2 database, we compared tumor tissues from TCGA with normal tissues from GTEx and found that SRD5A1, FUT8, and HES6 were significantly overexpressed in tumor tissues. By integrating data from immunohistochemical analysis of colorectal cancer patient tissues and colorectal cancer disease staging from the HPA database, we selected FUT8 as the target gene for further study (Fig 2A–C).

### 3.2. FUT8 expression with cellular functions

**Fig 3:**
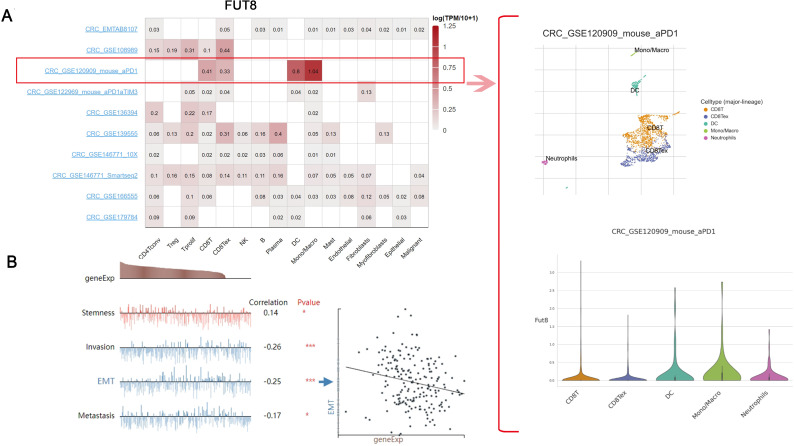
Expression of FUT8 at the cellular level and its correlation with cellular functions. A: Single-cell expression of FUT8 in different CRC cohorts. B: Potential correlation of FUT8 with four cellular functions in CRC cells.

In CRC tumor tissues, FUT8 was found to be enriched in CD8T cells and CD8Tex cells. However, in a cohort of CRC mice treated with PD-1 therapy, FUT8 was predominantly enriched in dendritic cells and mono/macrophage cells (Fig 3A). This suggests a potential correlation between this gene and tumor immunity. It is worth noting that the correlation of FUT8 with cellular functions varies across different cancers (S2 Table). In CRC cells, this gene was found to be significantly correlated with four cellular functions: stemness (Correlation 0.14, *P* = 0.03), invasion (Correlation -0.26, *P* < 0.001), EMT (Correlation -0.25, *P* < 0.001) and metastasis (Correlation -0.17, *P* < 0.01) (Fig 3B).

### 3.3. Tumor mutation burden

**Fig 4:**
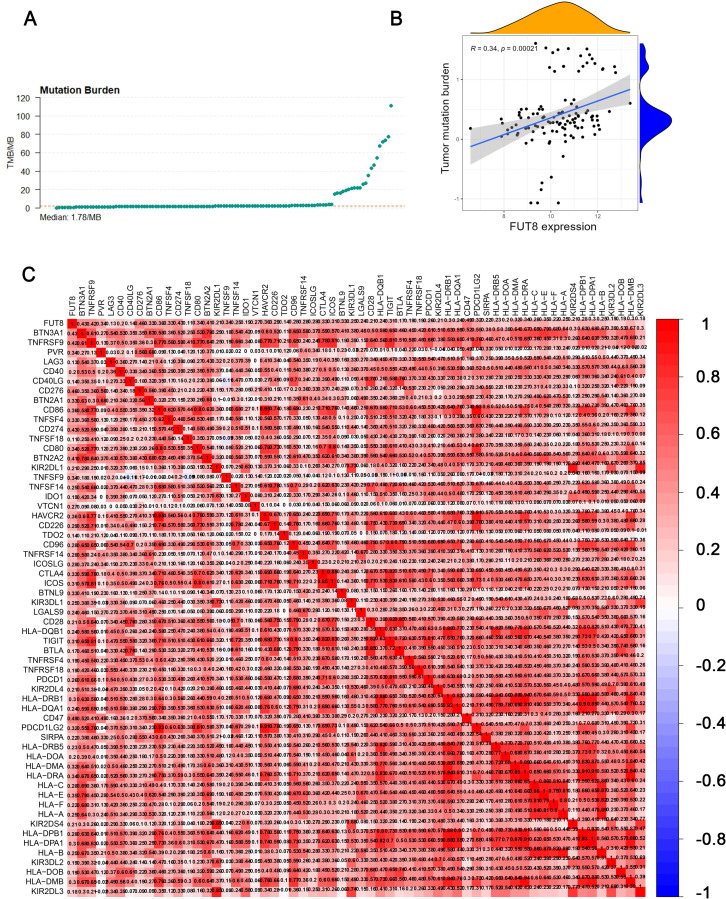
Correlation of FUT8 with tumor mutation burden and immune checkpoints. A: Tumor mutation burden scoring in the TCGA CRC dataset. B: Study on the correlation of FUT8 with tumor mutation burden. C: Co-expression analysis of FUT8 with 79 immune checkpoint genes.

The TCGA database was used to download the sample sheet data matrix for CRC, along with the corresponding mutation annotation format (MAF) data for each sample. The maftools package in R was used to perform immune load scoring, and TMB values were calculated (Fig 4A). The analysis of mutations in the CRC cohort showed that higher expression levels of FUT8 were associated with higher scores of tumor mutation burden (R = 0.34, *P* = 0.00021) (Fig 4B).

### 3.4. Immune checkpoint analysis

The analysis conducted co-expression of FUT8 with 79 immune checkpoint genes (S3 Table). Positive correlation between the checkpoint gene and the target gene is indicated by red color, while negative correlation is represented by blue color. Within the significant threshold range, a total of 59 immune checkpoint genes were found to be correlated with FUT8. The five immune checkpoint genes with the strongest correlation were CD47, CD276, CD274, BTN3A1, and TNFRSF9 (Fig 4C). This indicates that patients with CRC and high expression of FUT8 may be more responsive to immune therapy.

### 3.5. Immune infiltration

**Fig 5:**
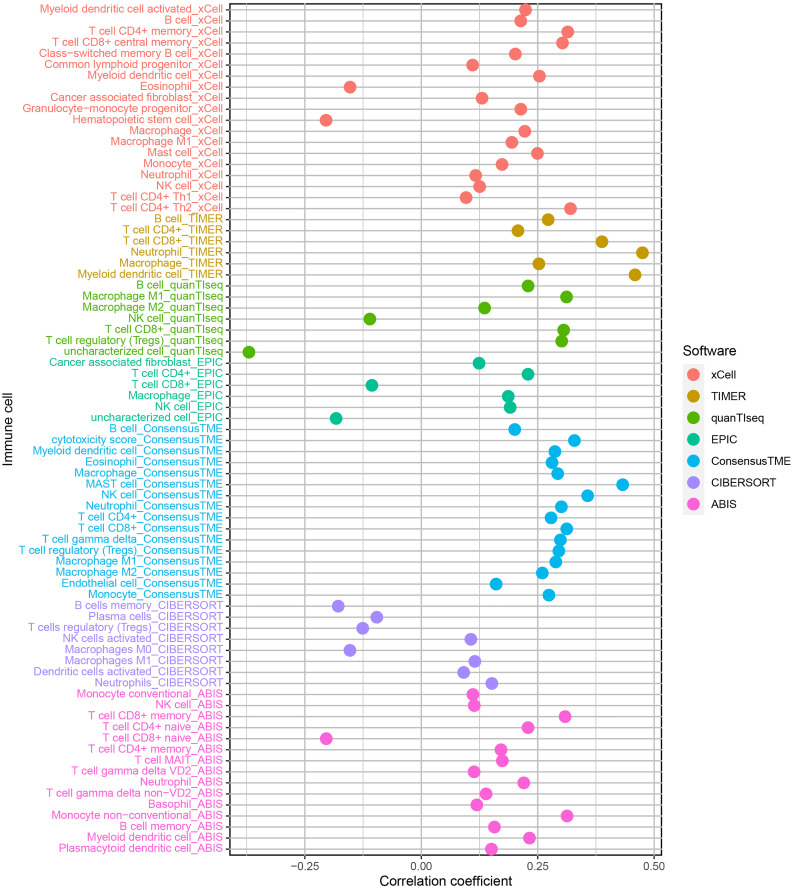
Impact of FUT8 expression on immune infiltration in CRC.

Based on published single-cell RNA sequencing studies of colorectal cancer (CRC), the tumor microenvironment was predominantly composed of epithelial cells (48.60%), followed by T/NK cells (18.68%), B cells (13.02%), endothelial cells (9.14%), myeloid cells (5.45%), and fibroblasts (5.11%) and other cells [15]. To investigate the role of FUT8 in immune regulation, we analyzed its correlation with immune cell infiltration. Using a stringent threshold (cor > 0.4, P < 0.05), FUT8 expression exhibited significant positive correlations with mast cells, neutrophils, and myeloid dendritic cells (Fig 5 and S4 Table). Myeloid dendritic cells bridge innate and adaptive immunity by activating T cells, a process that may be co-opted by FUT8 to facilitate immune evasion. These findings highlight FUT8 as a potential regulator that can influence the progression of immune subsets, playing a role in influencing immunity through a cascade caused by mast cells, neutrophils, and myeloid dendritic cells.

### 3.6. Molecular docking

**Fig 6:**
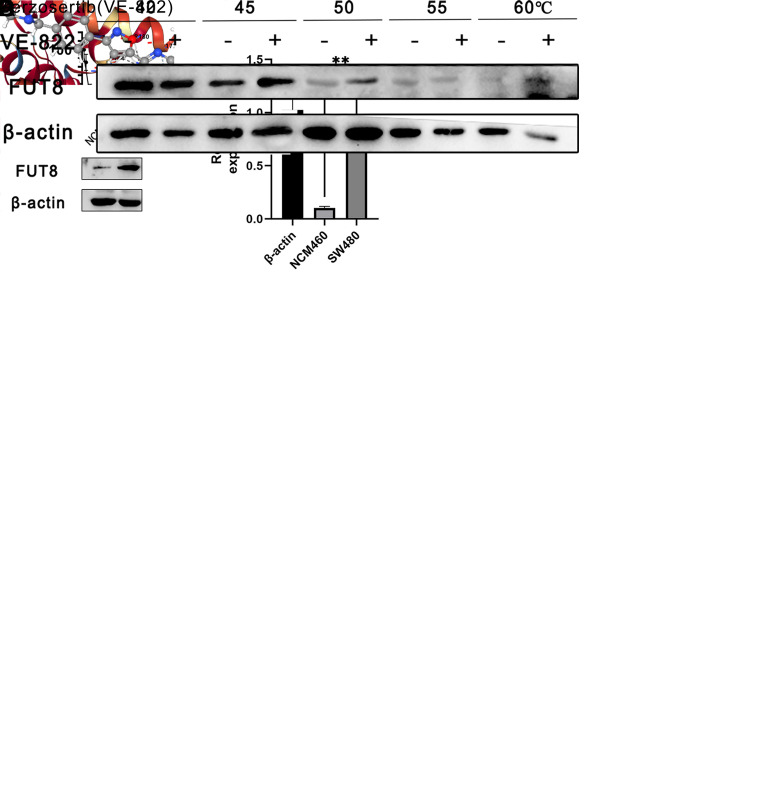
Experimental validation of the core targets. A: Molecular docking results of FUT8 with VE-822. B: Chemical structure of VE-822. C: Inhibitory effect of VE-822 on colon cancer cells. D: Differences in FUT8 between normal and colon cancer cells. E: The binding ability of VE-822 to FUT8 was verified by cellular thermal shift assay. *P < 0.05; **P < 0.01, ***P < 0.001.

Molecular docking was performed using the drugs as ligands and FUT8 as the protein receptor (S1–S21 Figs and Table 1). The docking binding energy were compared to determine the optimal intervening drug, VE-822 (Fig 6A).

**Table 1:** Molecular docking results of FUT8 with compounds.

Gene	Drug	Vina score
FUT8	pyridoclax	‒8.8
FUT8	Imatinib	‒8.7
FUT8	trametinib	‒8.1
FUT8	Mebendazole	‒8.4
FUT8	Alpelisib	‒8.8
FUT8	Alectinib	‒8.3
FUT8	Vemurafenib	‒8.0
FUT8	Dabrafenib	‒8.9
FUT8	Ibrutinib	‒8.2
FUT8	Abemaciclib	‒8.6
FUT8	Copanlisib	‒8.4
FUT8	Axitinib	‒8.6
FUT8	Lenvatinib	‒8.3
FUT8	Sorafenib	‒9.0
FUT8	Sunitinib	‒7.9
FUT8	Ribavirin	‒6.0
FUT8	DMAPT	‒7.0
FUT8	Colchicine	‒7.7
FUT8	auranofin	‒6.9
FUT8	**VE-822**	‒**9.2**
FUT8	Brivanib	‒7.9

### 3.7. Cell proliferation

VE-822, Berzosertib, is an ATR inhibitor that inhibits chemotherapy-induced DNA damage in non-small cell lung cancer (NSCLC) cells (Fig 6B). The MTT assay, a widely used method for measuring cell viability and proliferation, demonstrated that VE-822 exhibited a dose-dependent inhibitory effect on SW480 cells. The IC_50_ value of 1.245 μM indicates the concentration at which VE-822 is able to inhibit the growth of SW480 cells by 50% (Fig 6C). These findings suggest that VE-822 has potential as an anti-cancer agent for targeting SW480 cells and may warrant further investigation for its therapeutic applications in cancer treatment.

### 3.8. Differential expression of FUT8 in tumor cells and normal cells

In order to investigate the differences in FUT8 protein expression between normal colon cells and tumor cells, NCM460 cells and SW480 cells were selected for culturing in this study. The total protein of both cell lines was extracted and analyzed to compare the levels of FUT8 expression. The results revealed a significant difference in FUT8 expression between the two cell lines, with tumor cells showing a notably higher level of FUT8 protein compared to normal colon cells (Fig 6D). This finding suggests that increased FUT8 expression may be associated with tumorigenesis in colon cells, highlighting the potential significance of targeting FUT8 as a therapeutic strategy for colorectal cancer.

### 3.9. Cellular thermal shift assay

Cellular thermal shift assay (CETSA) is a method to detect the interaction between drugs and target proteins directly in cells or tissues [16]. The basic principle is that after ligand and protein binding, the thermal stability of the protein will be improved to a certain extent, based on which the ability to bind to the ligand can be verified. In this study, cells were intervened with 0.5 μM VE-822, proteins were extracted after 48 hours, and protein samples were heated at five temperatures. We observed that FUT8 protein was less affected by heat in cells treated with VE-822 (Fig 6E).

## 4. Discussion

The FUT8 gene is located on chromosome 14q23.3 and encodes an enzyme that catalyzes core fucosylation, a necessary N-glycan modification [17–19]. Compared to other FUT family proteins, the FUT8 protein possesses an SH3 domain, which is a key residue for FUT8 activity and is unique to FUT8 [20]. In recent years, studies have found that the expression of FUT8 is associated with the occurrence and development of various tumors and can modulate the expression of tumor-related factors. The invasiveness of breast cancer cells and their invasion and metastasis can be stimulated by FUT8 through the remodeling of TGF-β receptor core fucosylation [18]. In hepatocellular carcinoma (HCC) cells, HCC development can be promoted by the circRNA cFUT8 through binding to free miR-548c and inhibiting the miR-548c/FUT8 axis [21]. FUT8-AS1 and its downstream feedback loop are involved in the development of oral squamous cell carcinoma by activating the Wnt/β-catenin signaling pathway [22]. Interestingly, FUT8 was also found to have an impact on the prognosis of p53 status in stage II and III CRC, as observed by Masaru Noda et al [23]. Furthermore, a recent study showed that FUT8 mediates nuclear fucosylation of the key immune checkpoint molecule (ICM) CD276 (B7-H3) in CRC. The first FUT8-targeting inhibitor developed by the research team showed significant efficacy in metastatic CRC [24]. Researchers have also discovered that suppressing FUT8 through genetic ablation or pharmacological inhibition can reduce surface expression of PD-1 and enhance T-cell activation, thereby more effectively eradicating tumors [25].

Previous studies have shown that FUT8 has great potential for targeted tumor therapy, colon cancer staging, and the tumor immune environment. Genetic data analyses that we have performed are consistent with these findings to some extent. The discovery of eQTLs provides valuable insights into the molecular mechanisms underlying complex traits and diseases. They help to identify specific proteins that act as mediators between genotype and phenotype [5,26–29]. Transcriptional analysis at the single-cell level can provide evidence for mechanisms of disease progression at the genetic level [30–33]. This research suggests that FUT8 inhibitors may have a potential protective effect in CRC patients. However, our study has some limitations. The majority of subjects in this study were of European ancestry, and this causal relationship may differ in other ethnic groups. Alternatively, despite the study setting’s efforts to avoid error, weak instrument bias may arise in MR and lead to weak relationships. Therefore, extensive biological research and experimental validation are needed before their clinical application. In addition, more comprehensive analysis and data mining are required to investigate whether similar clinical relevance and tumor immune effects of FUT8 are exhibited in CRC cohorts beyond the dataset used in this study.

## 5. Conclusion

Through the integration of computational modeling and experimental validation, this study has not only identified VE-822 as a promising candidate for targeted therapy of CRC but has also provided deeper insights into the biological mechanisms underlying FUT8-mediated tumorigenesis. These findings are expected to inspire future drug development efforts aimed at leveraging the therapeutic potential of targeting FUT8 in the field of oncology.

## Supporting information

S1 Table The eQTLs causally associated with CRC discovered by Mendelian randomization. (XLSX)

S2 Table The correlation of FUT8 with cellular functions in different cancers. (XLSX)

S3 Table 79 immune checkpoint genes used in gene expression correlations. (XLSX)

S4 Table Correlation analysis between the expression of FUT8 and immune infiltration. (XLSX)

S5 Table Finn R9 summary data. (XLSX)

S1 Fig Molecular docking diagram of FUT8 and pyridoclax. (TIF)

S2 Fig Molecular docking diagram of FUT8 and Imatinib. (TIF)

S3 Fig Molecular docking diagram of FUT8 and trametinib. (TIF)

S4 Fig Molecular docking diagram of FUT8 and Mebendazole. (TIF)

S5 Fig Molecular docking diagram of FUT8 and Alpelisib. (TIF)

S6 Fig Molecular docking diagram of FUT8 and Alectinib. (TIF)

S7 Fig Molecular docking diagram of FUT8 and Vemurafenib. (TIF)

S8 Fig Molecular docking diagram of FUT8 and Dabrafenib. (TIF)

S9 Fig Molecular docking diagram of FUT8 and Ibrutinib. (TIF)

S10 Fig Molecular docking diagram of FUT8 and Abemaciclib. (TIF)

S11 Fig Molecular docking diagram of FUT8 and Copanlisib. (TIF)

S12 Fig Molecular docking diagram of FUT8 and Axitinib. (TIF)

S13 Fig Molecular docking diagram of FUT8 and Lenvatinib. (TIF)

S14 Fig Molecular docking diagram of FUT8 and Sorafenib. (TIF)

S15 Fig Molecular docking diagram of FUT8 and Sunitinib. (TIF)

S16 Fig Molecular docking diagram of FUT8 and Ribavirin. (TIF)

S17 Fig Molecular docking diagram of FUT8 and DMAPT. (TIF)

S18 Fig Molecular docking diagram of FUT8 and Colchicine. (TIF)

S19 Fig Molecular docking diagram of FUT8 and auranofin. (TIF)

S20 Fig Molecular docking diagram of FUT8 and VE-822. (TIF)

S21 Fig Molecular docking diagram of FUT8 and Brivanib. (TIF)

S1 File Images of Wb experiments showing the effect of compounds on the expression of target proteins. (DOCX)

S2 File R program code and environment configuration. (DOCX)

S1 STROBE MR Checklist STROBE MR checklist. (DOCX)

S1 Data Raw data of cell viability of FUT8 cells were measured by MTT assay (1-1). (XLS)

S2 Data Raw data of cell viability of FUT8 cells were measured by MTT assay (1-2). (XLS)

S3 Data Raw data of cell viability of FUT8 cells were measured by MTT assay (1-3). (XLS)

S4 Data Raw data of cell viability of FUT8 cells were measured by MTT assay (2-1). (XLS)

S5 Data Raw data of cell viability of FUT8 cells were measured by MTT assay (2-2). (XLS)

S6 Data Raw data of cell viability of FUT8 cells were measured by MTT assay (2-3). (XLS)

S7 Data Raw data of cell viability of FUT8 cells were measured by MTT assay (3-1). (XLS)

S8 Data Raw data of cell viability of FUT8 cells were measured by MTT assay (3-2). (XLS)

S9 Data Raw data of cell viability of FUT8 cells were measured by MTT assay (3-3). (XLS)
