# Supplementary figures and images for: Identification of hub gene associated with colorectal cancer: Integrating Mendelian randomization, transcriptome analysis and experimental verification
Source: PLoS Genet. 2025 Jul 29;21(7):e1011788. doi: 10.1371/journal.pgen.1011788 (PMC12349882; doi:10.1371/journal.pgen.1011788)

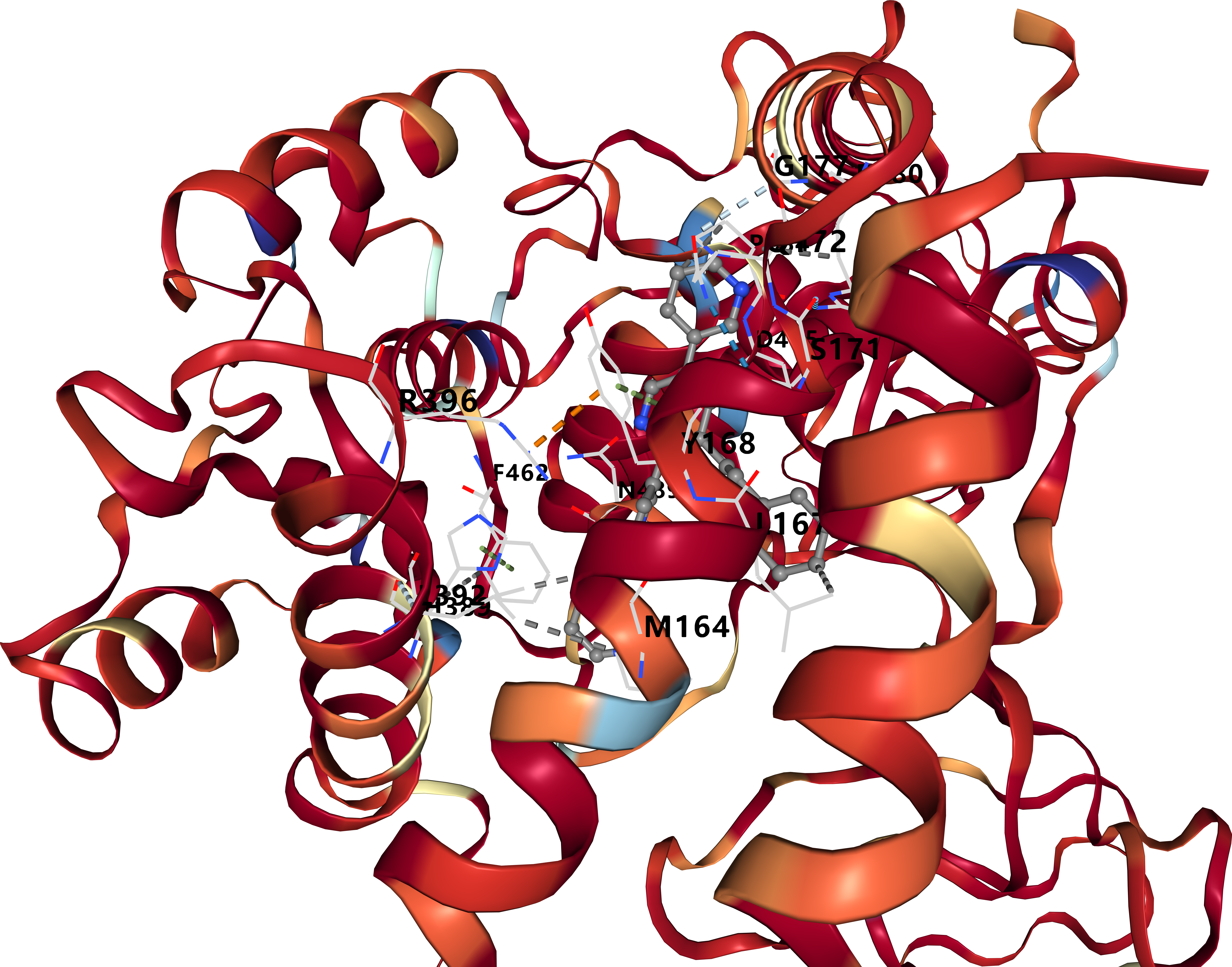

Supplement: S1 Fig — (TIF) [file pgen.1011788.s006.tif]

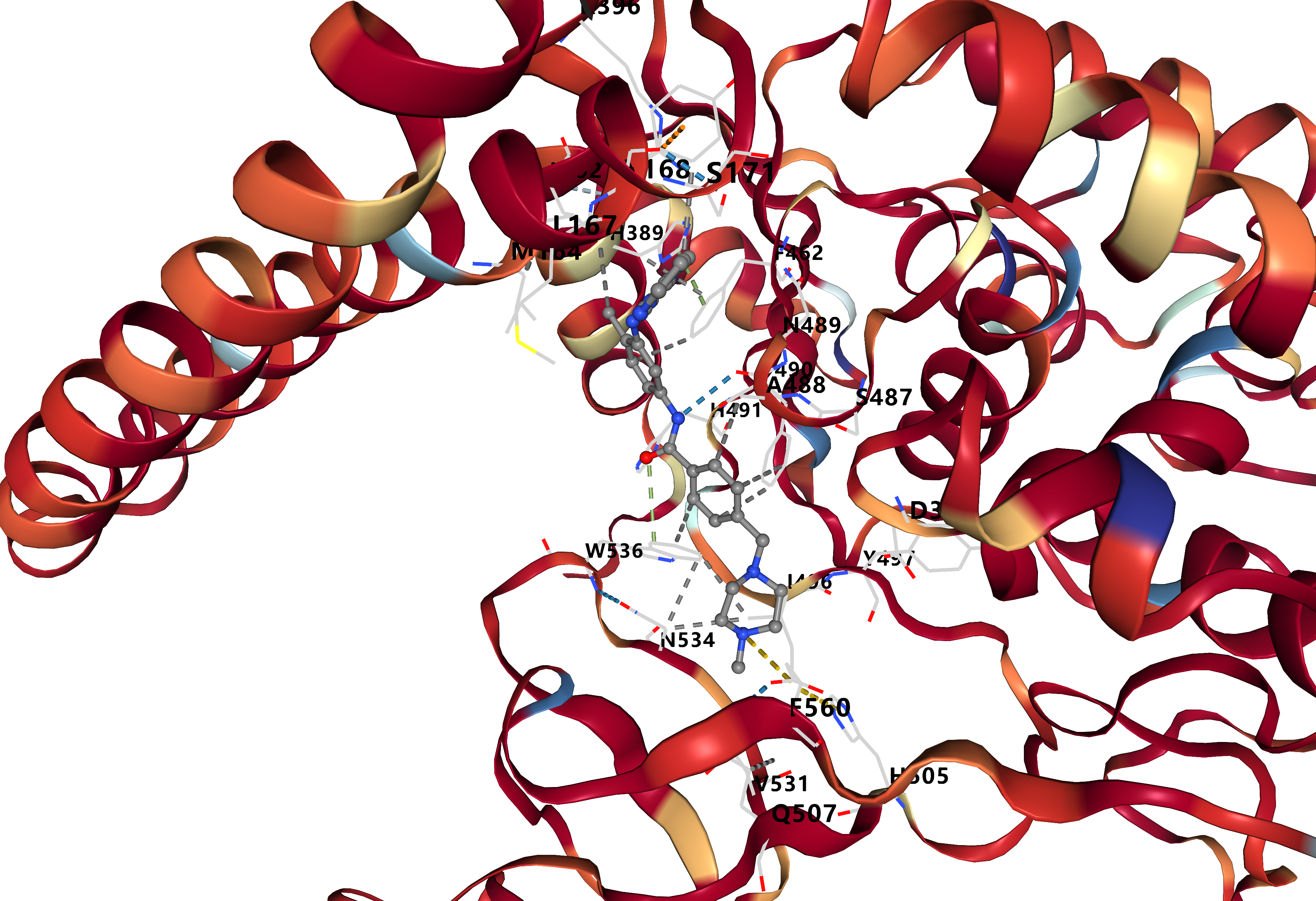

Supplement: S2 Fig — (TIF) [file pgen.1011788.s007.tif]

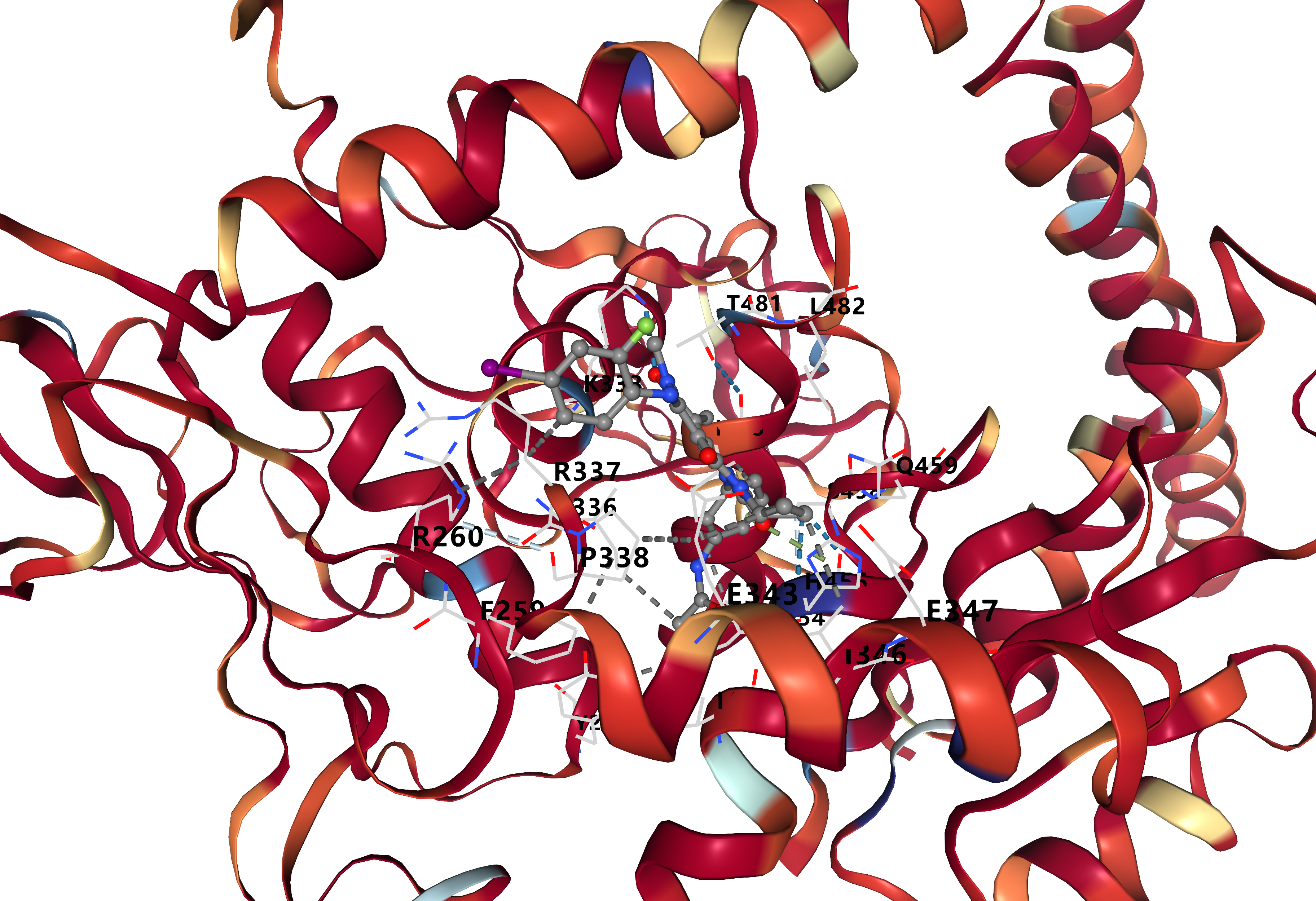

Supplement: S3 Fig — (TIF) [file pgen.1011788.s008.tif]

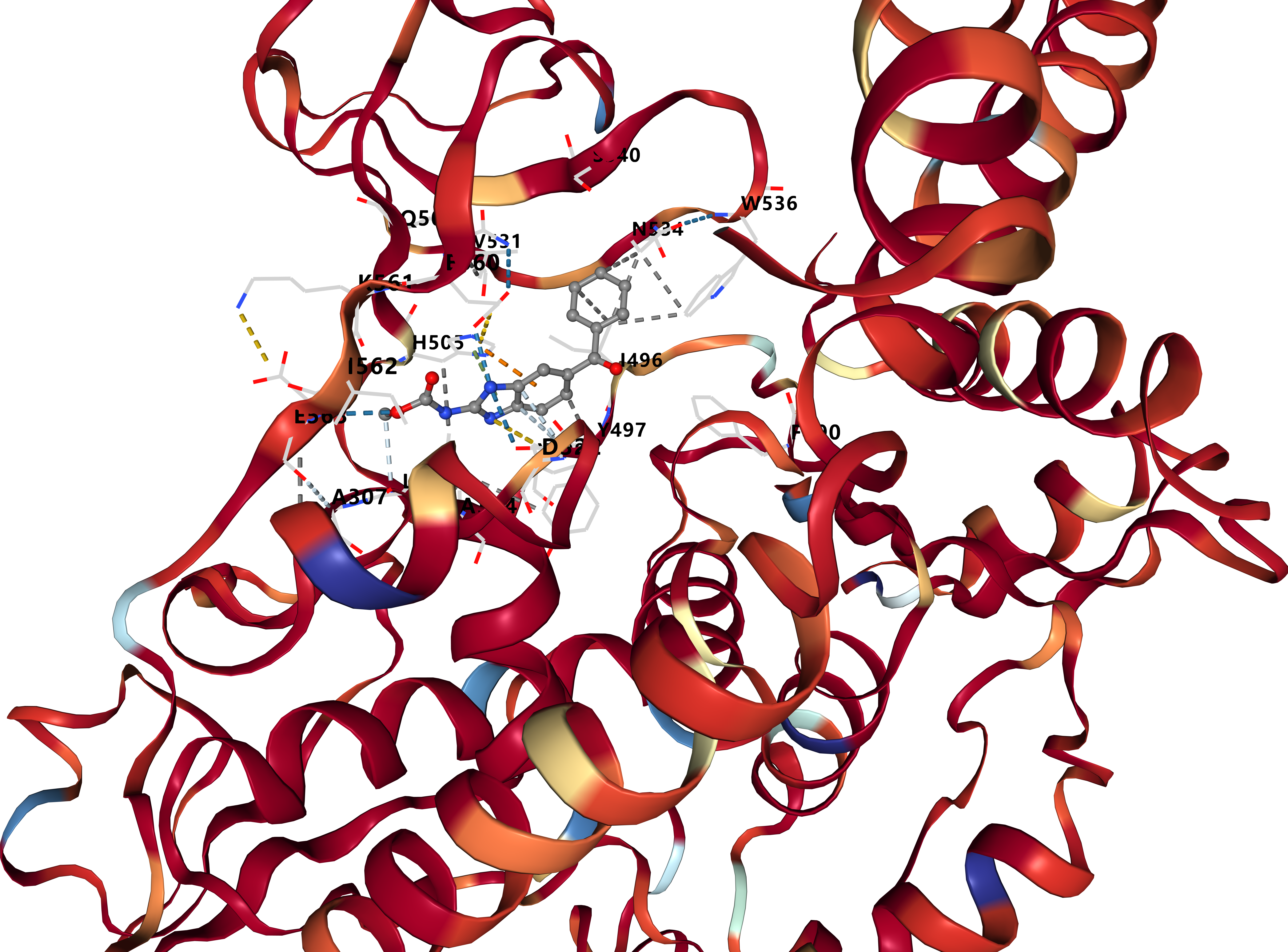

Supplement: S4 Fig — (TIF) [file pgen.1011788.s009.tif]

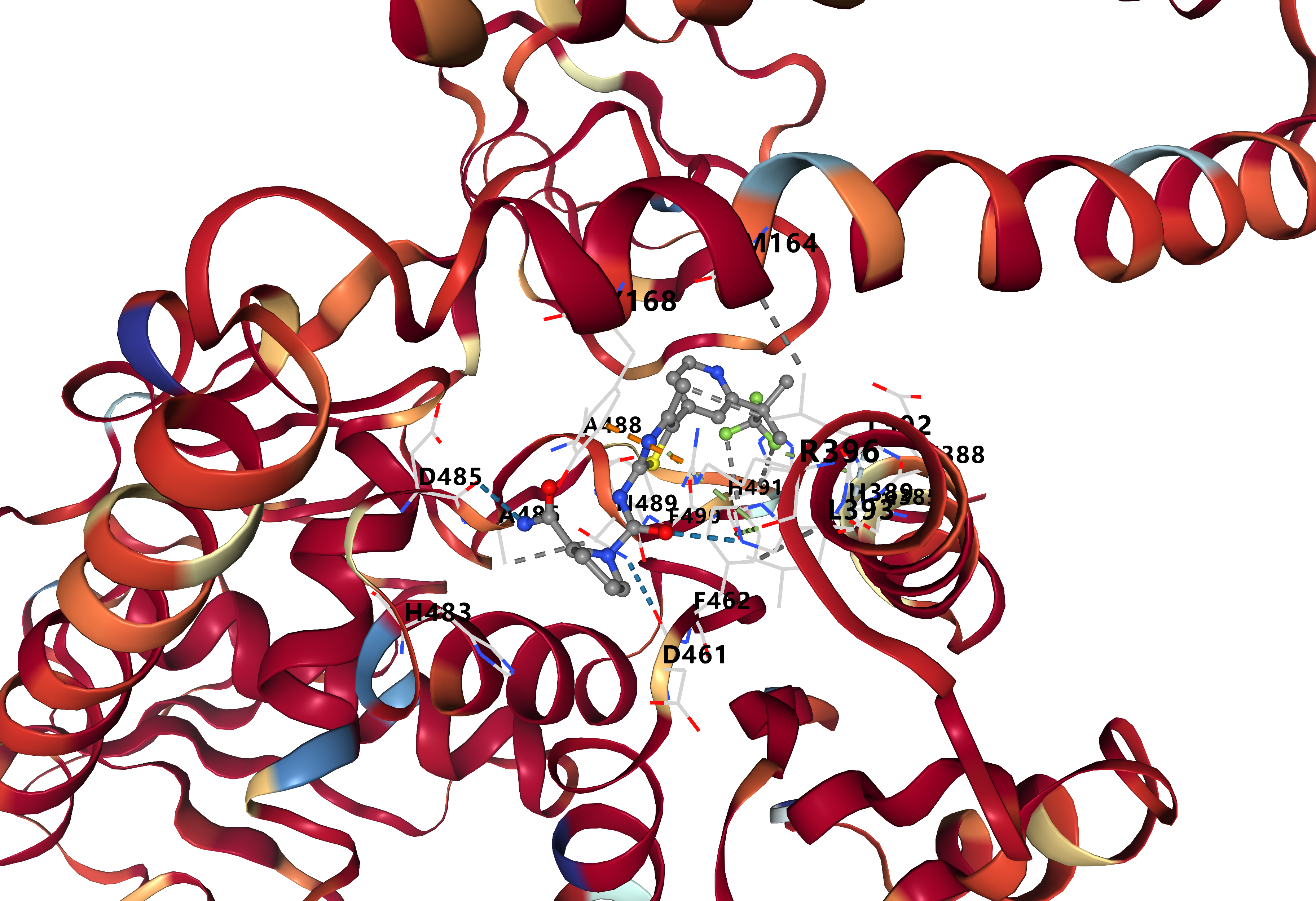

Supplement: S5 Fig — (TIF) [file pgen.1011788.s010.tif]

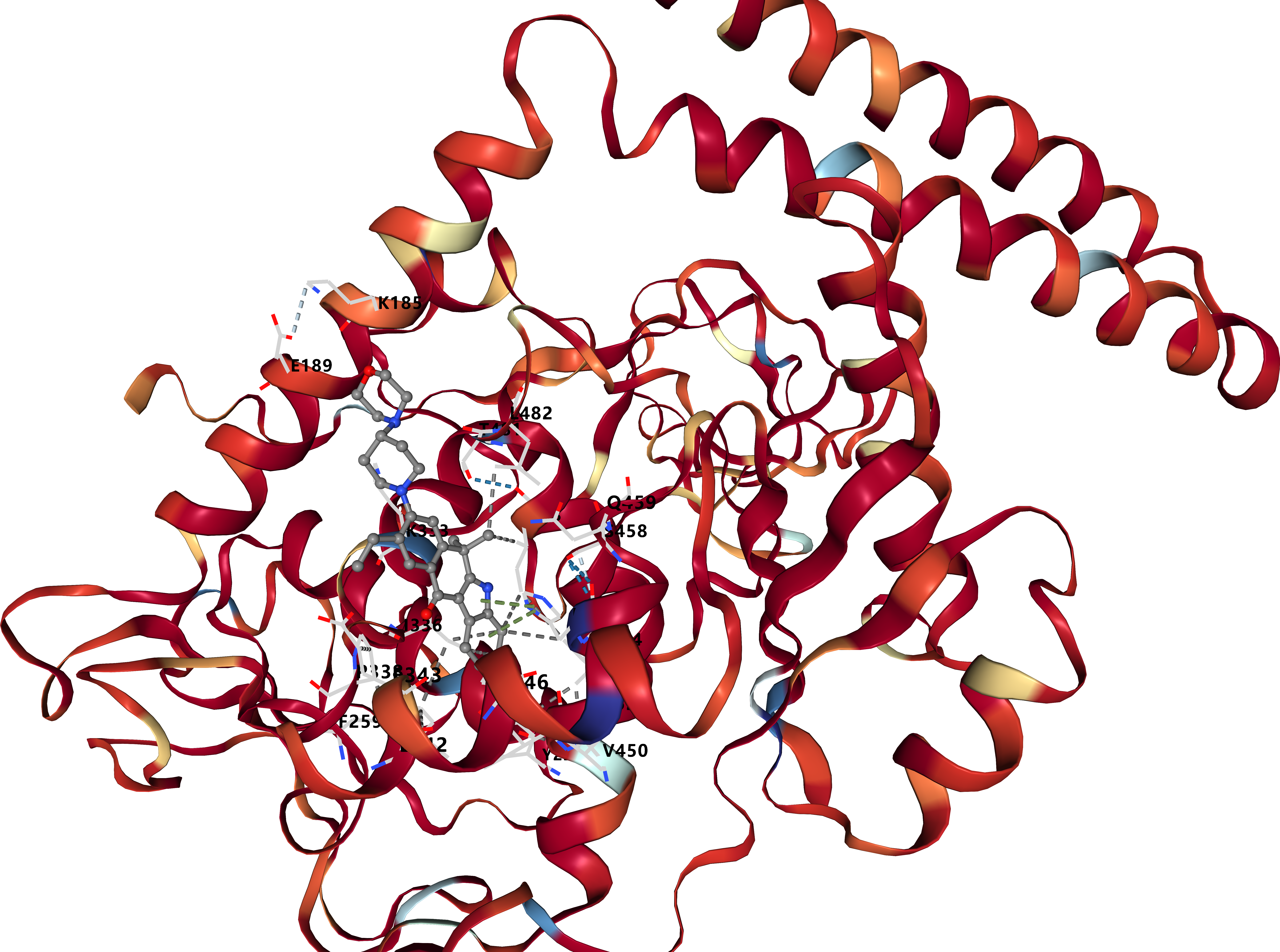

Supplement: S6 Fig — (TIF) [file pgen.1011788.s011.tif]

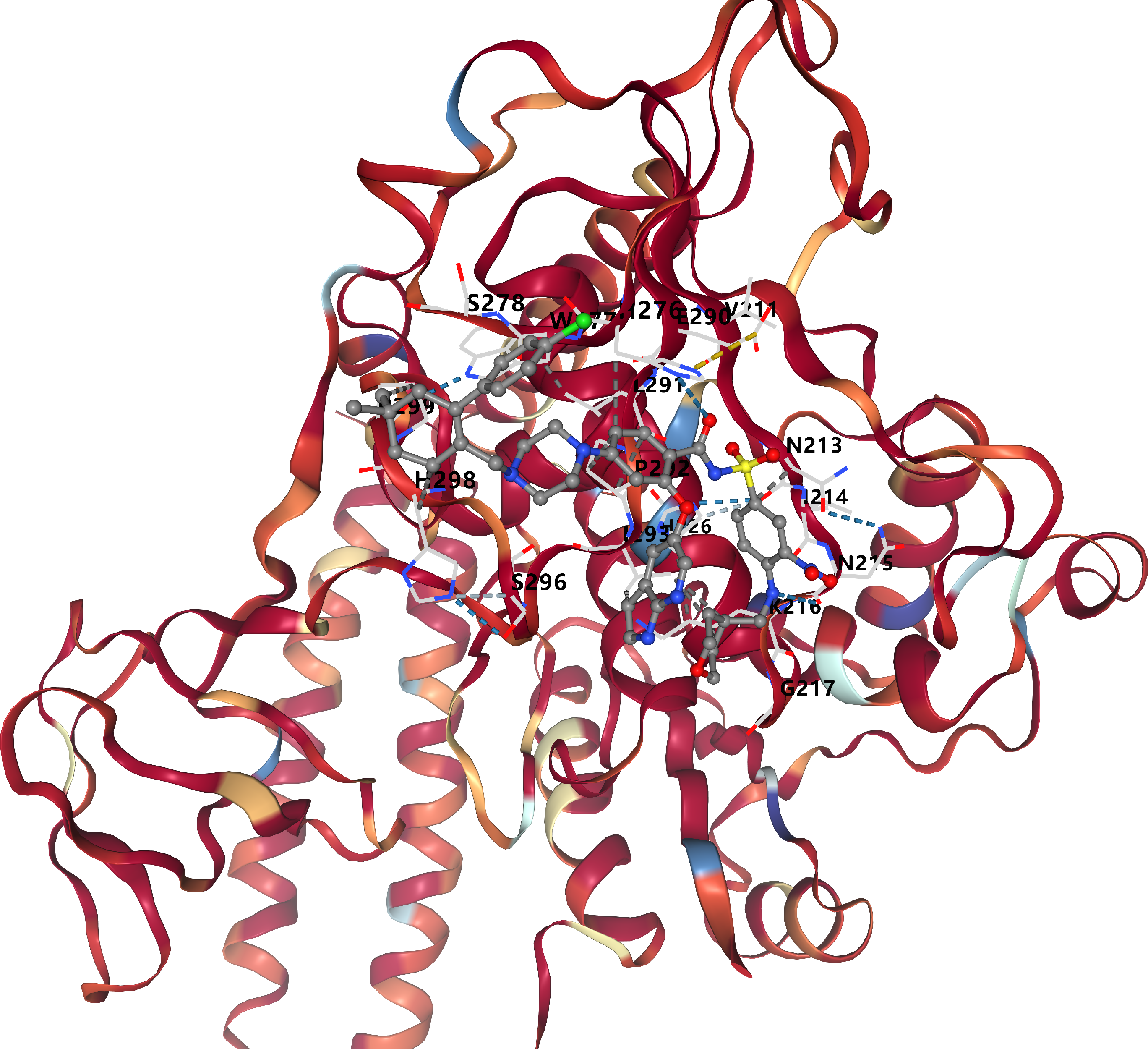

Supplement: S7 Fig — (TIF) [file pgen.1011788.s012.tif]

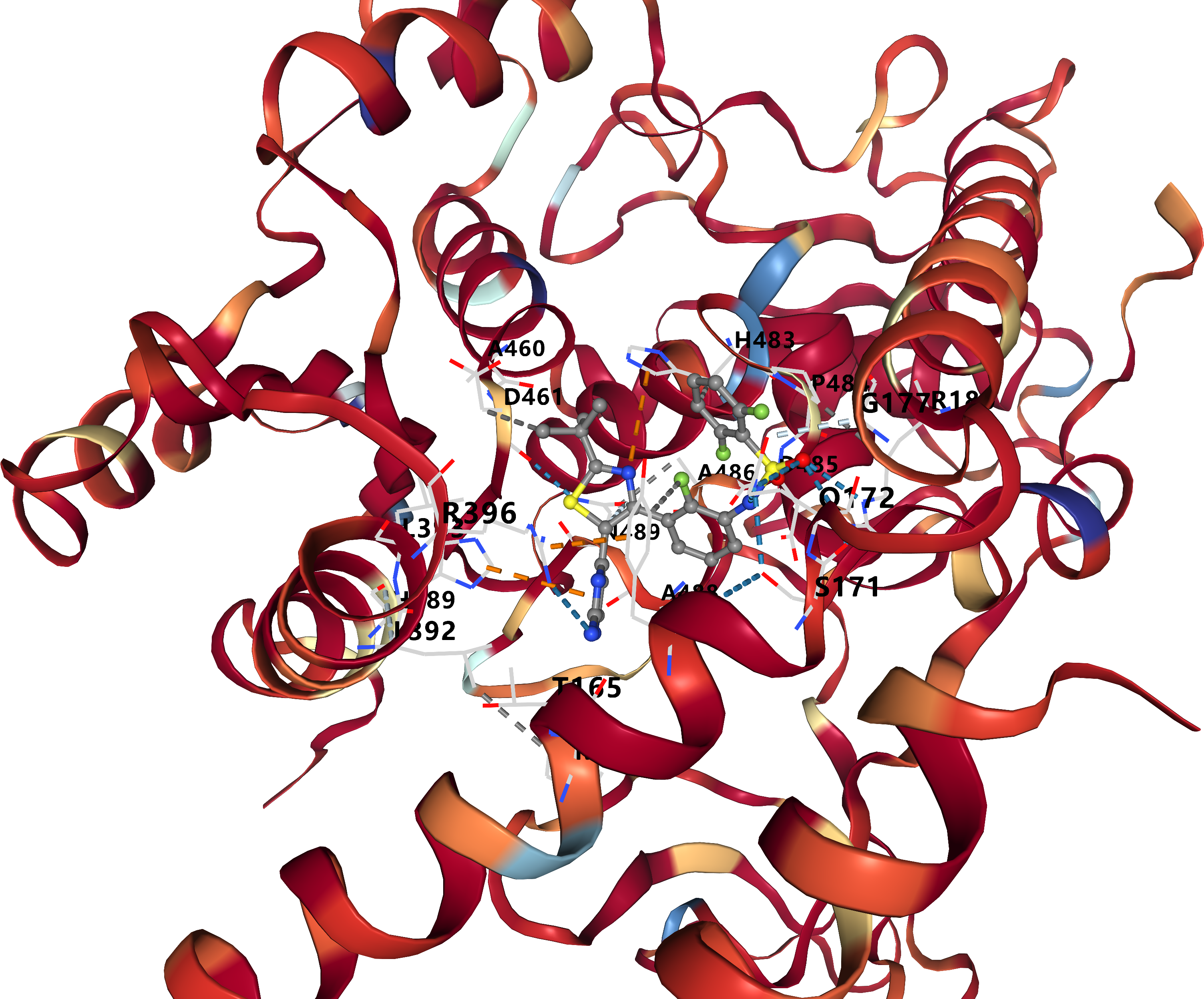

Supplement: S8 Fig — (TIF) [file pgen.1011788.s013.tif]

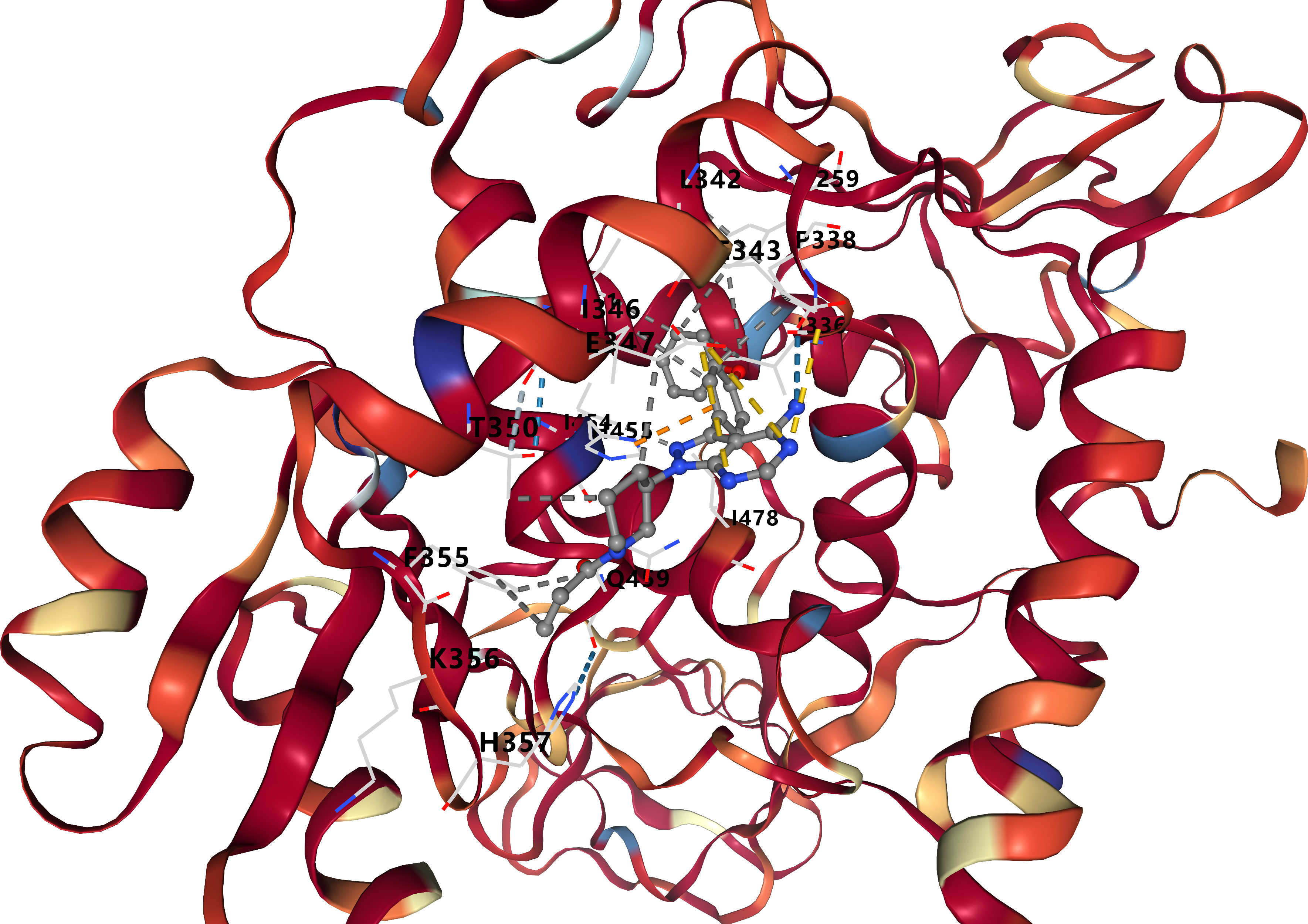

Supplement: S9 Fig — (TIF) [file pgen.1011788.s014.tif]

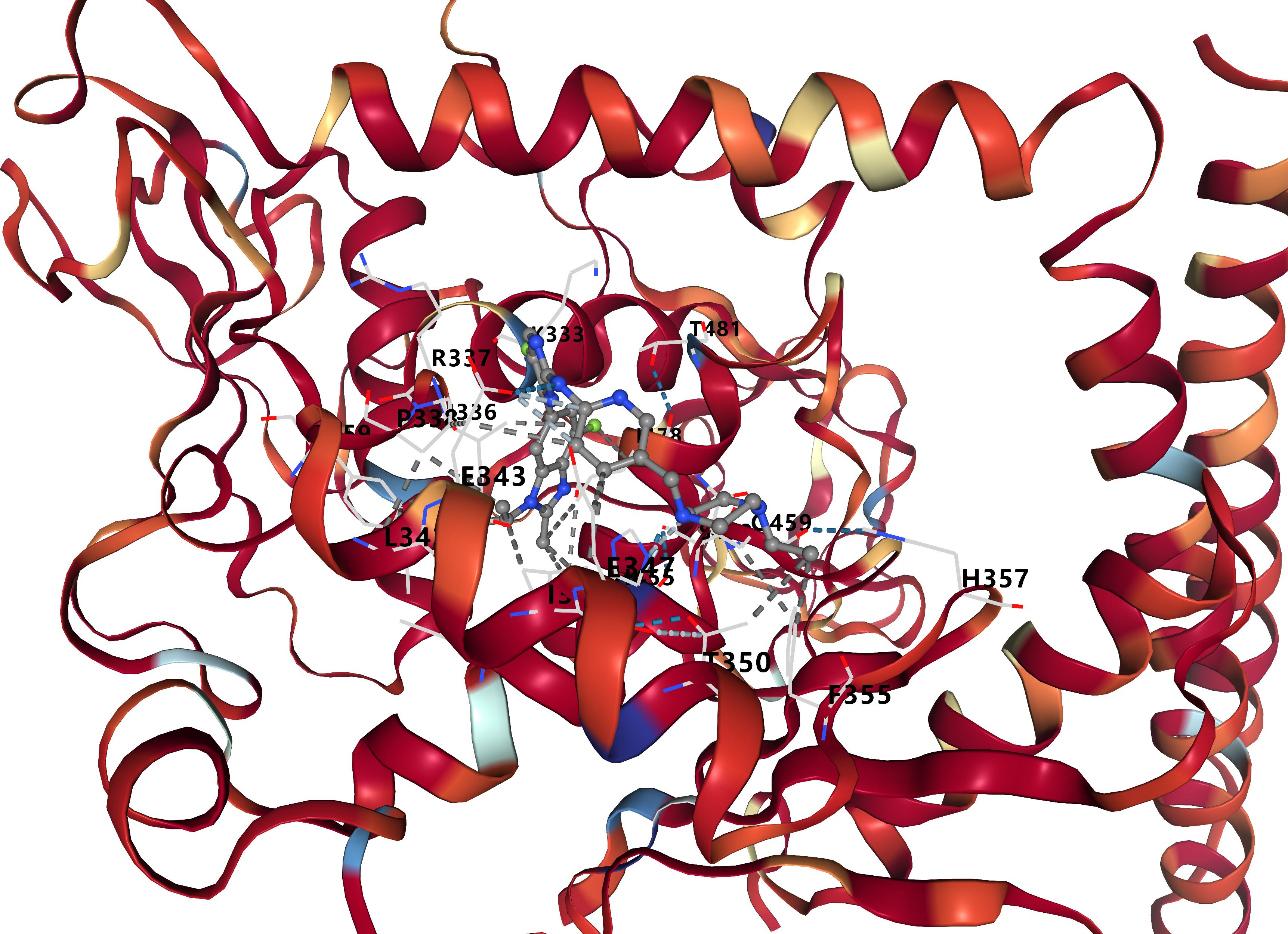

Supplement: S10 Fig — (TIF) [file pgen.1011788.s015.tif]

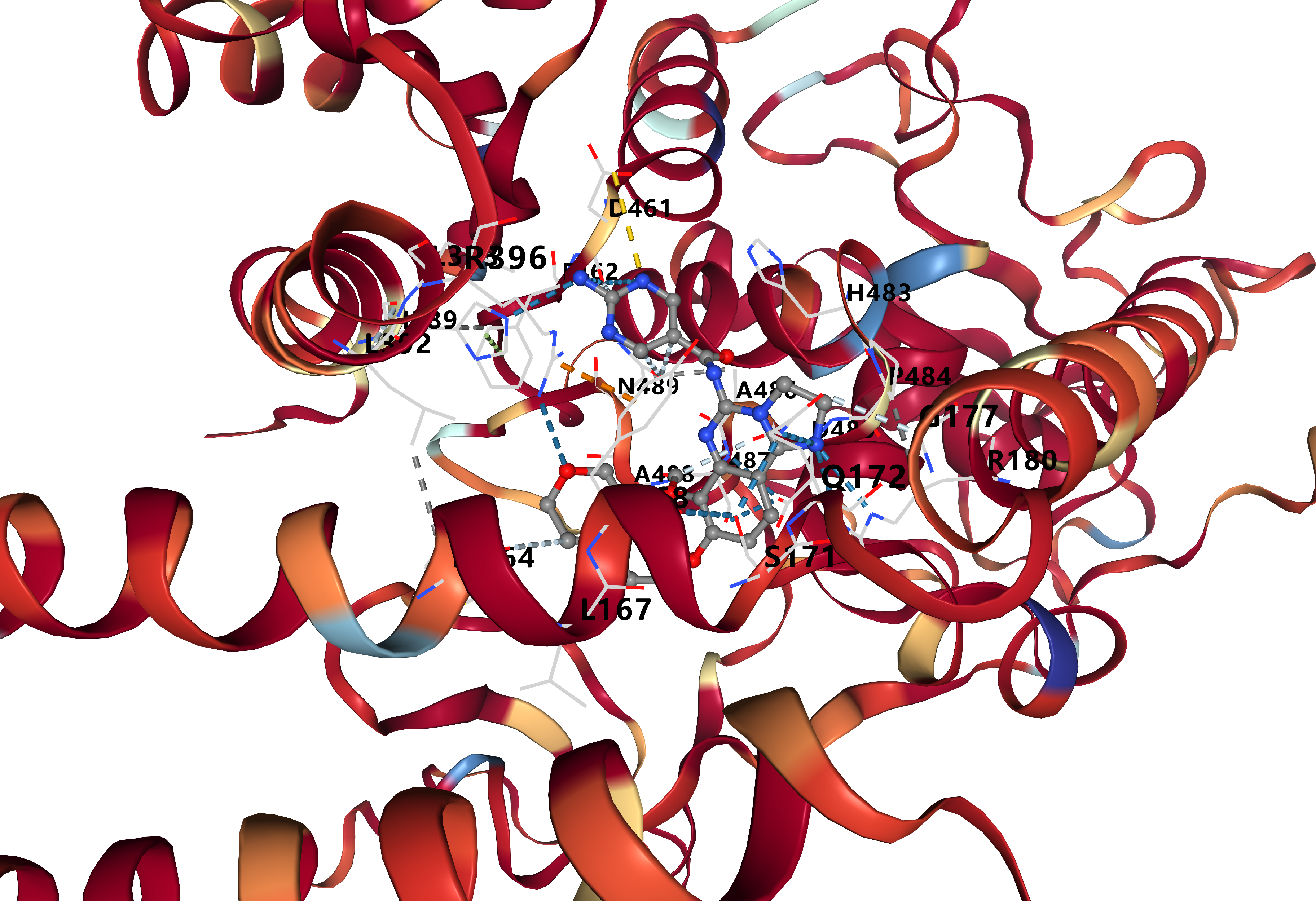

Supplement: S11 Fig — (TIF) [file pgen.1011788.s016.tif]

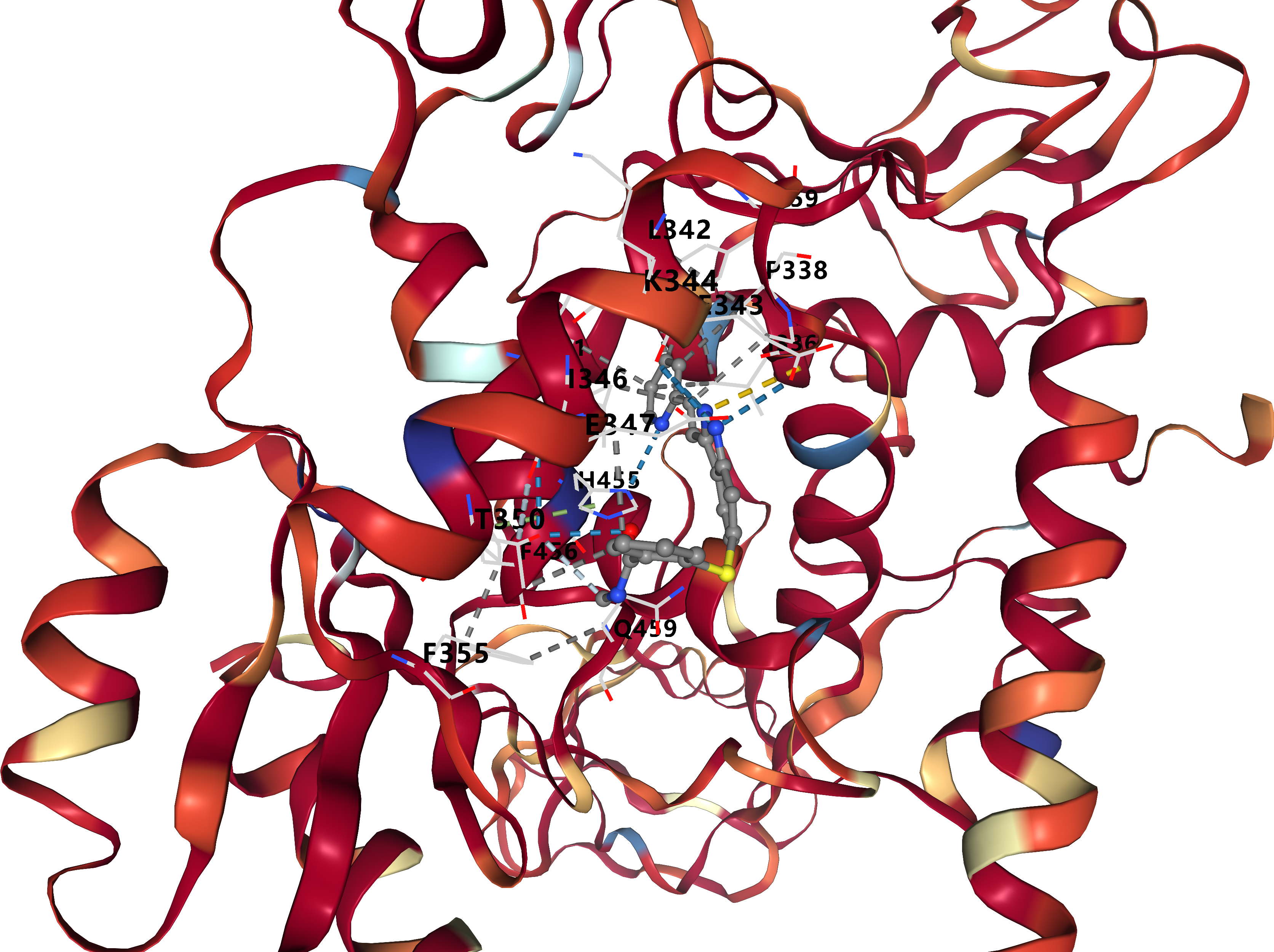

Supplement: S12 Fig — (TIF) [file pgen.1011788.s017.tif]

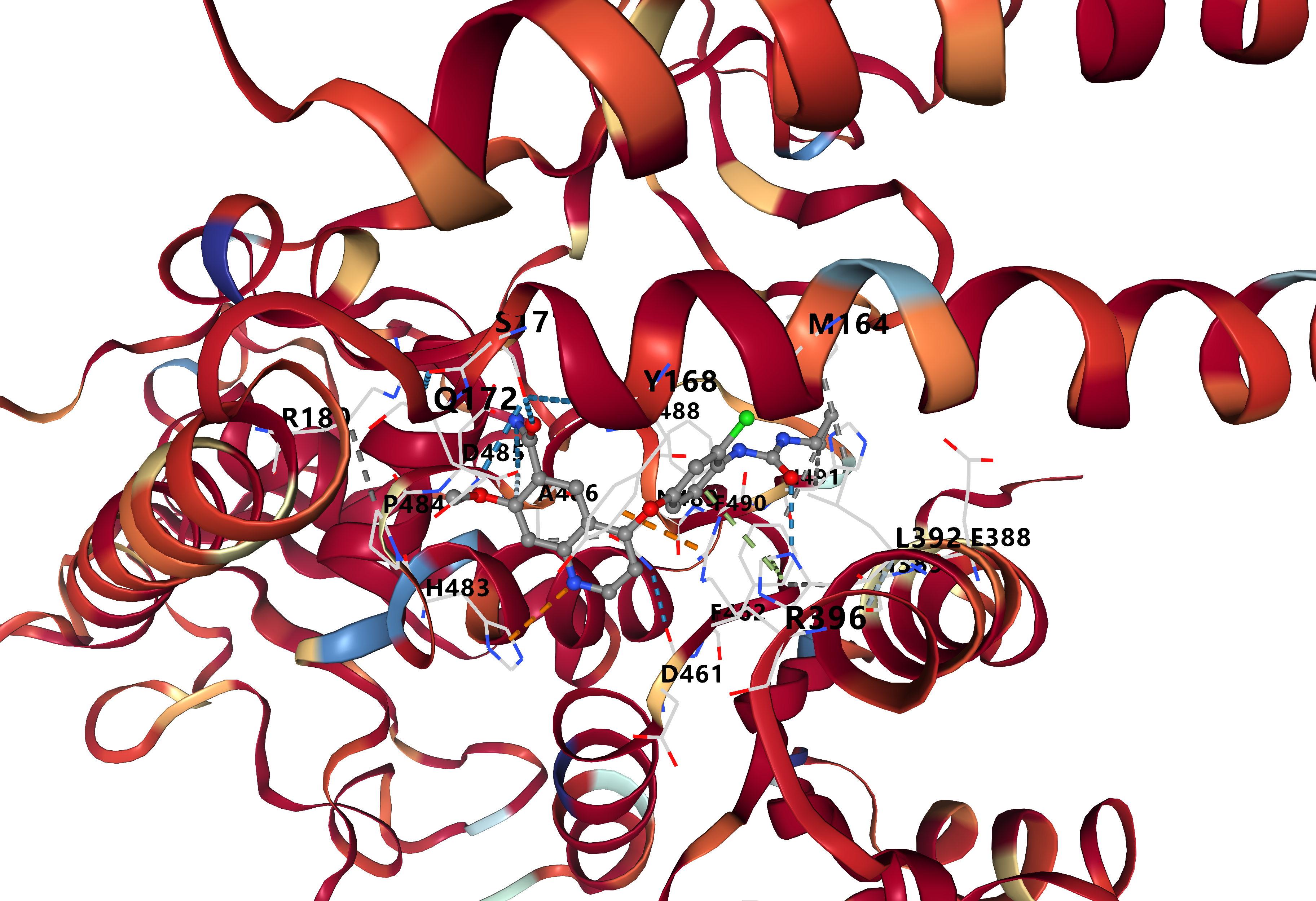

Supplement: S13 Fig — (TIF) [file pgen.1011788.s018.tif]

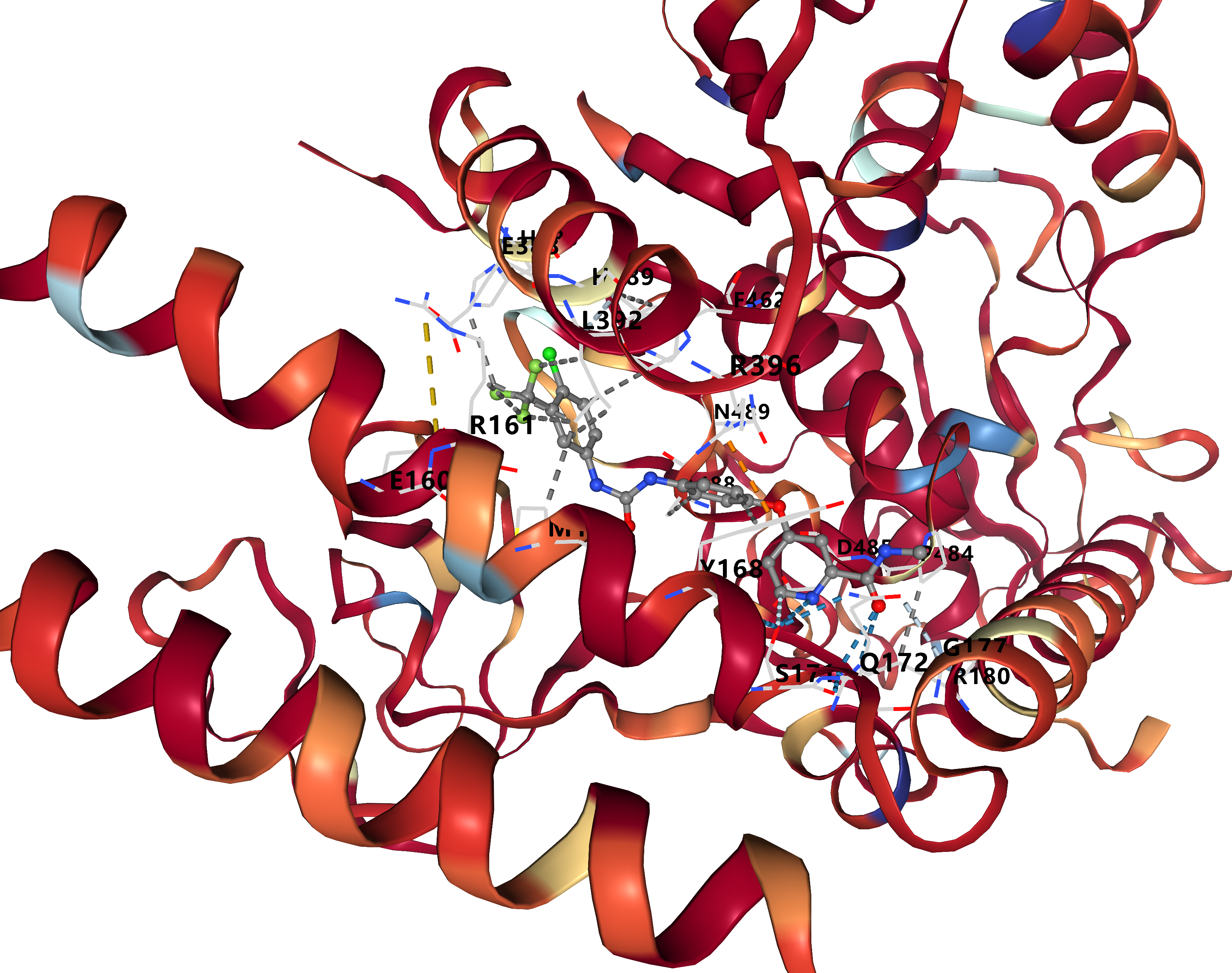

Supplement: S14 Fig — (TIF) [file pgen.1011788.s019.tif]

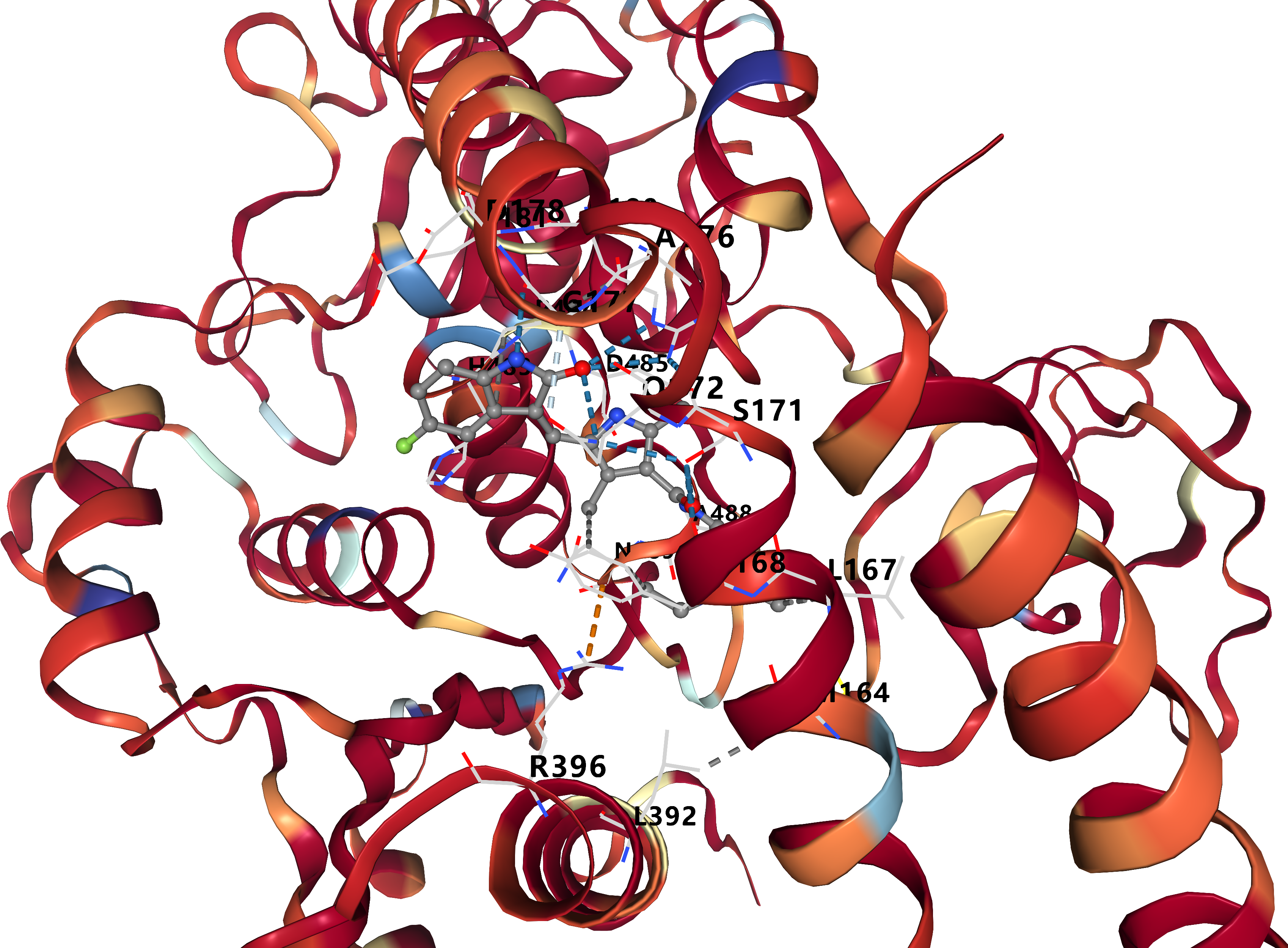

Supplement: S15 Fig — (TIF) [file pgen.1011788.s020.tif]

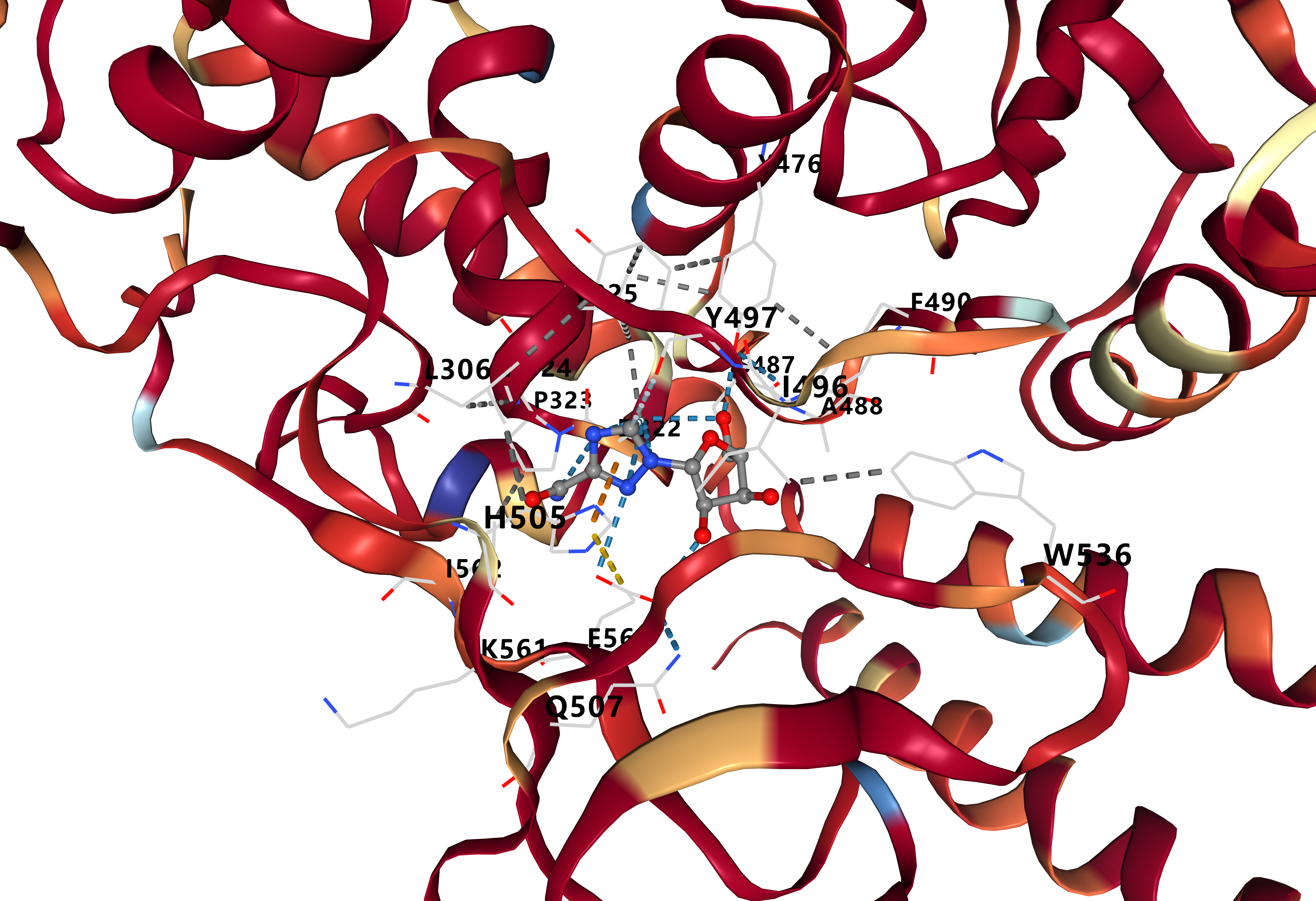

Supplement: S16 Fig — (TIF) [file pgen.1011788.s021.tif]

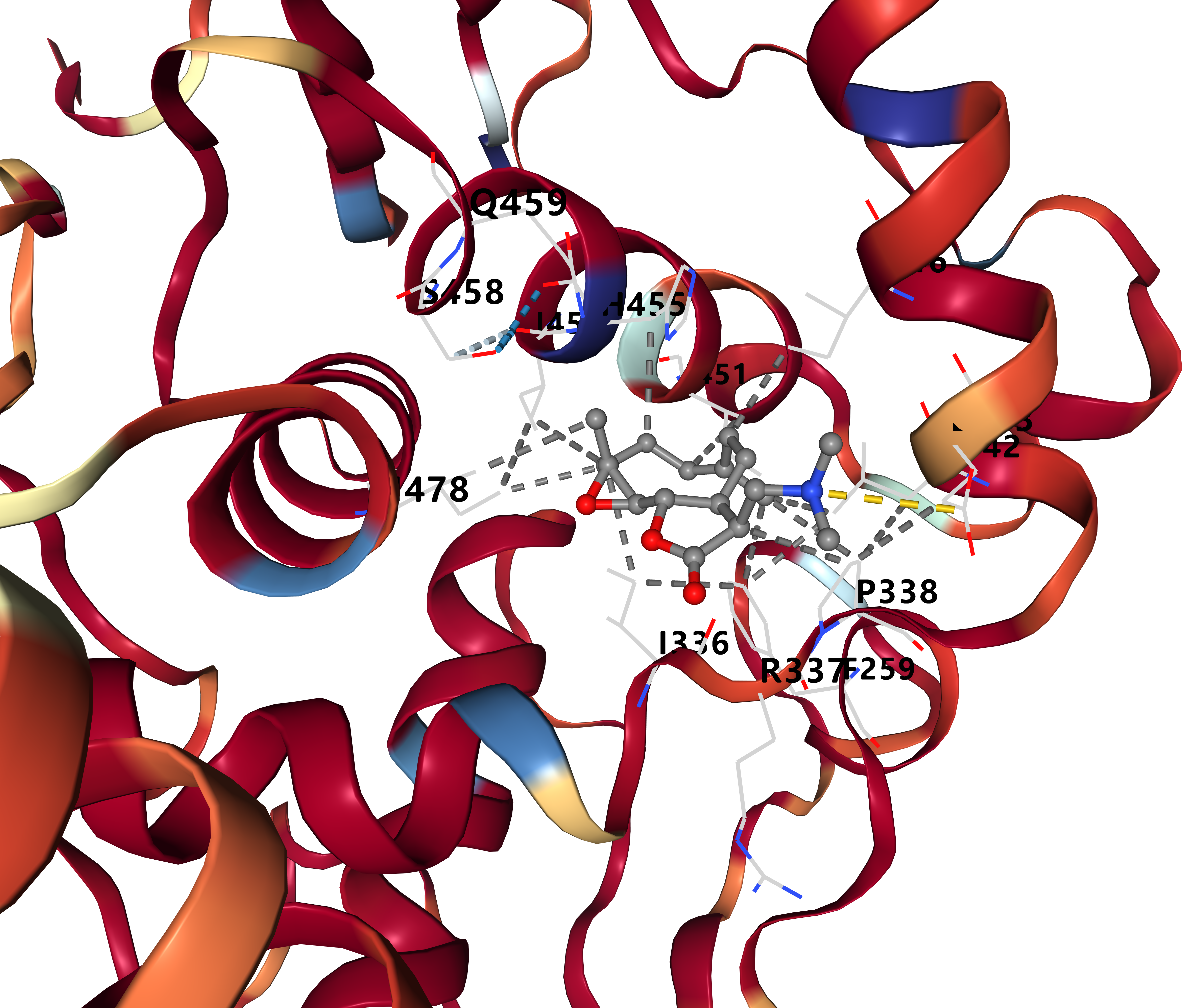

Supplement: S17 Fig — (TIF) [file pgen.1011788.s022.tif]

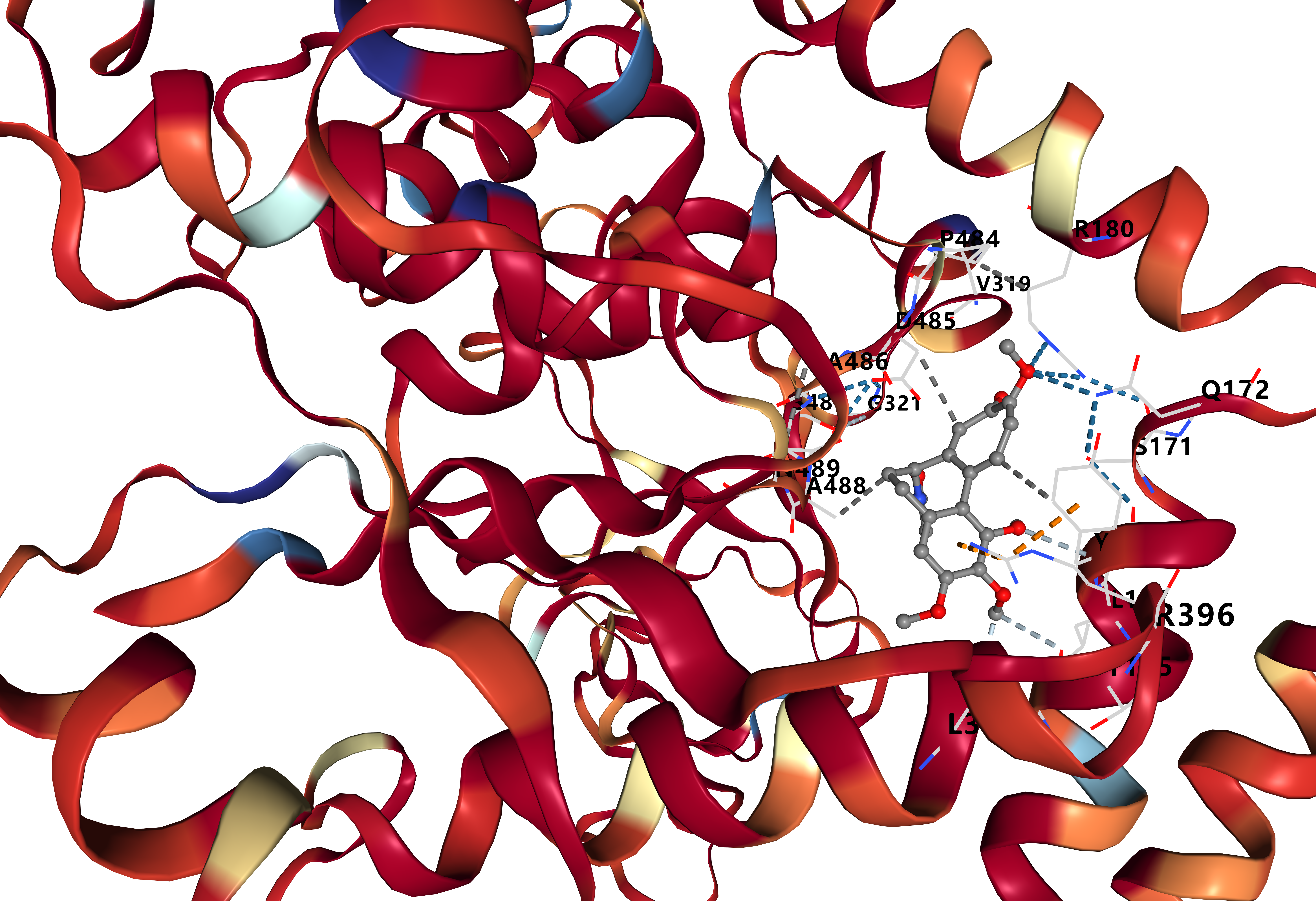

Supplement: S18 Fig — (TIF) [file pgen.1011788.s023.tif]

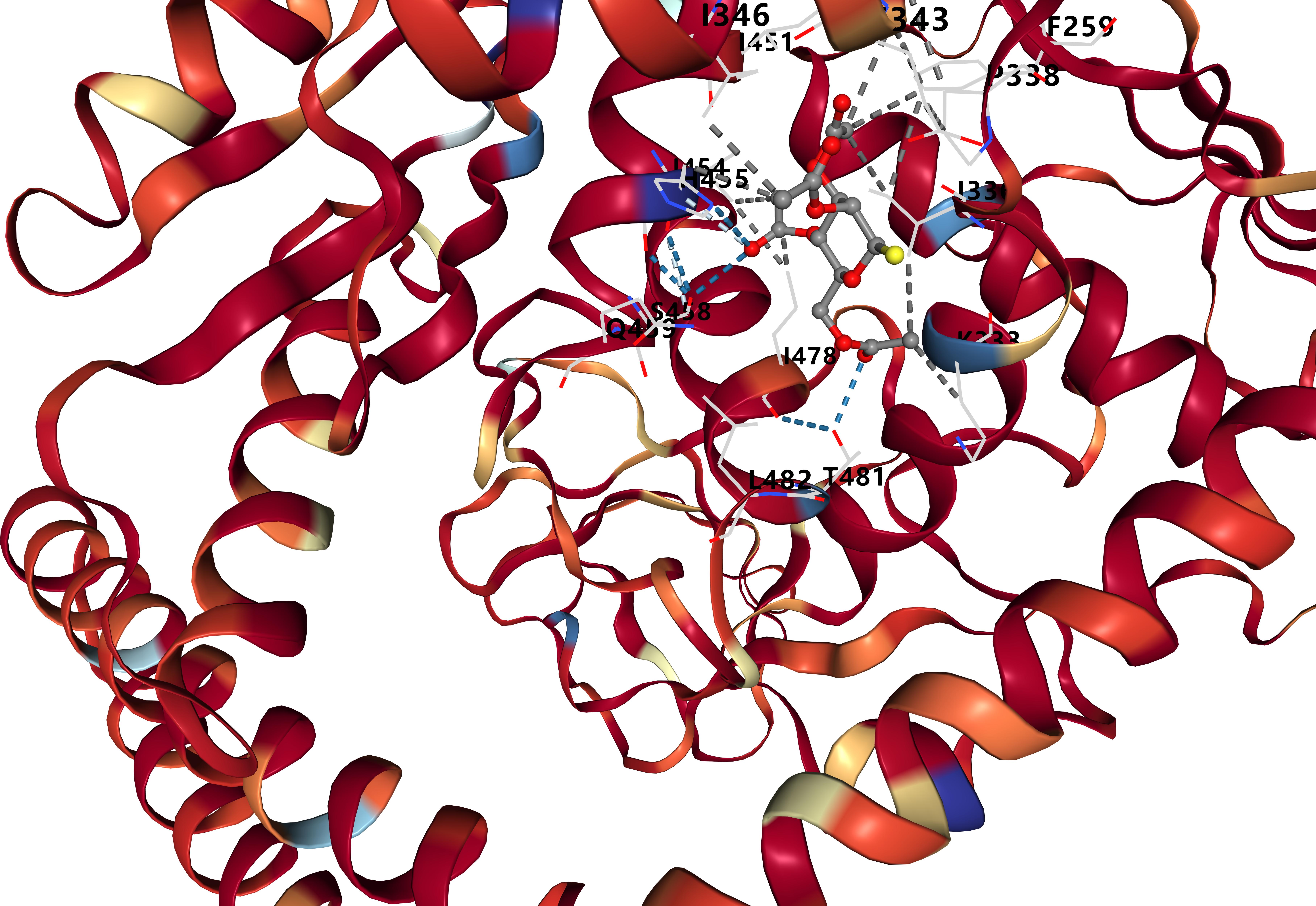

Supplement: S19 Fig — (TIF) [file pgen.1011788.s024.tif]

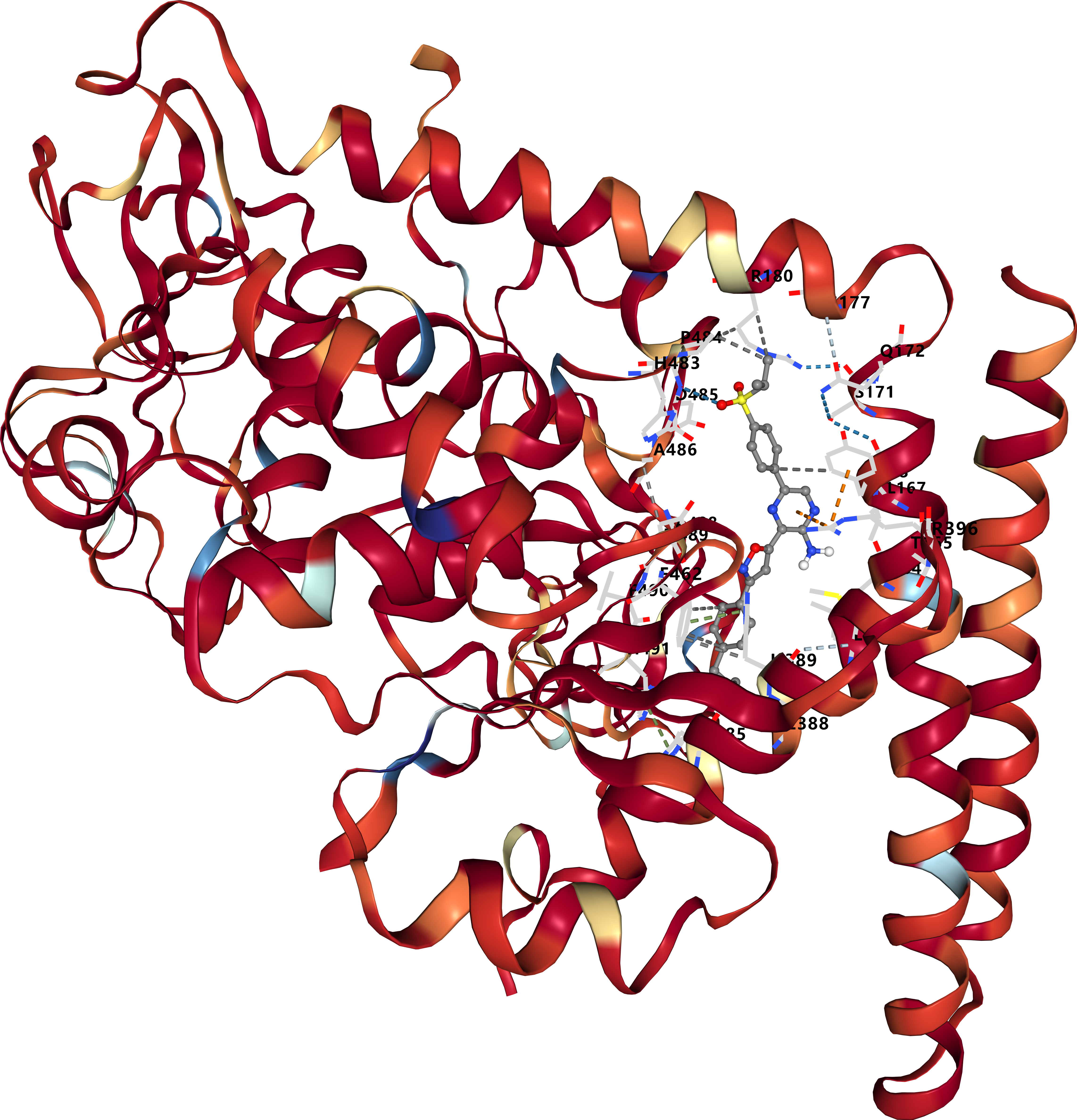

Supplement: S20 Fig — (TIF) [file pgen.1011788.s025.tif]
