# Supplementary material for: Identification of hub gene associated with colorectal cancer: Integrating Mendelian randomization, transcriptome analysis and experimental verification
Source: PLoS Genet. 2025 Jul 29;21(7):e1011788. doi: 10.1371/journal.pgen.1011788 (PMC12349882; doi:10.1371/journal.pgen.1011788)

Gene expression

Figure 9 C

FUT8 (66KD)

From left to right:

Marker, NCM460-1, SW480-1, NCM460-2, SW480-2, NCM460-3, SW480-3, Marker


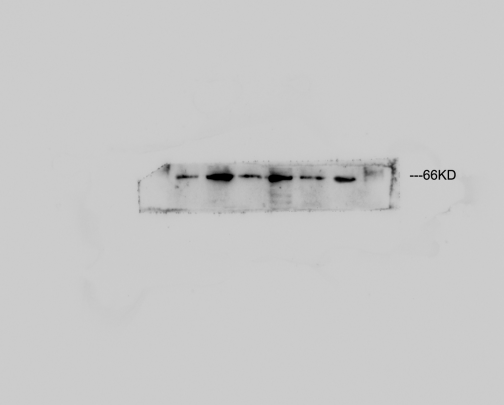


β-actin (42KD)

From left to right:

Marker, NCM460-1, SW480-1, NCM460-2, SW480-2, NCM460-3, SW480-3, Marker


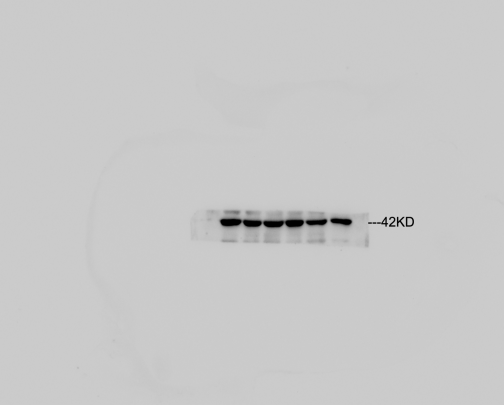


Cellular thermal shift assay

Figure 9 D

From left to right:

40 °C, 40 °C+VE-822，45 °C，45 °C+VE-822，50 °C，50 °C+VE-822，55 °C，55 °C+VE-822，60 °C，60 °C+VE-822

FUT8 (66KD)


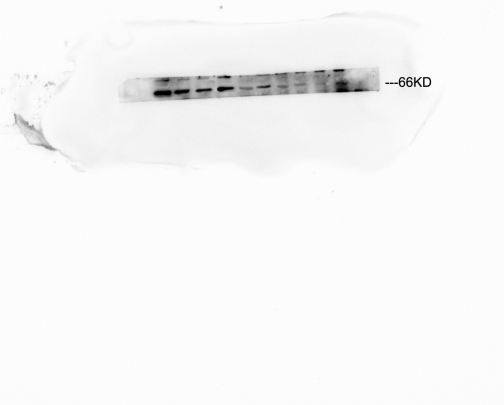


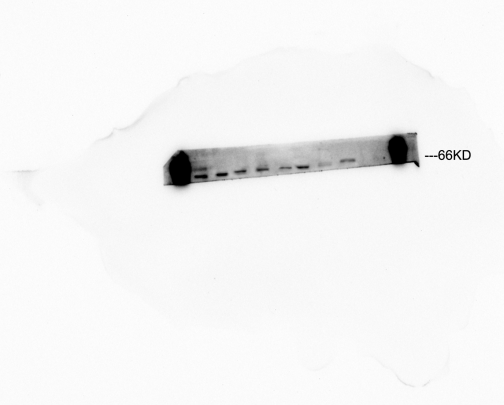


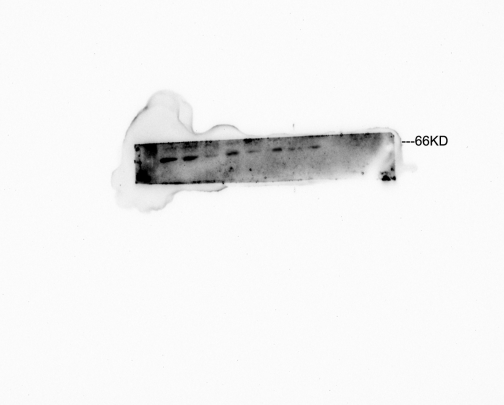


From left to right:

40 °C, 40 °C+VE-822，45 °C，45 °C+VE-822，50 °C，50 °C+VE-822，55 °C，55 °C+VE-822，60 °C，60 °C+VE-822

β-actin (42KD)


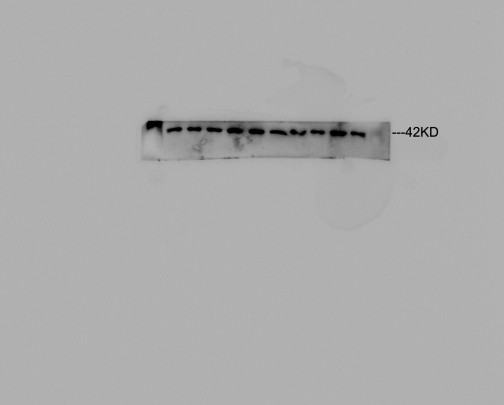


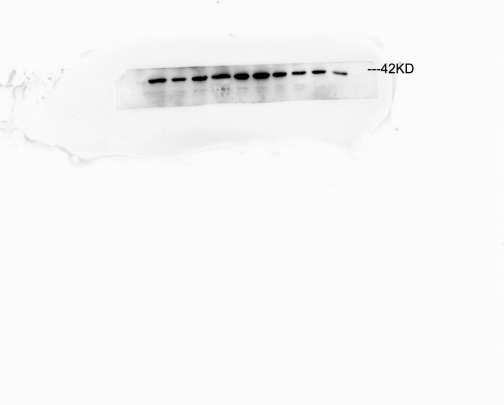


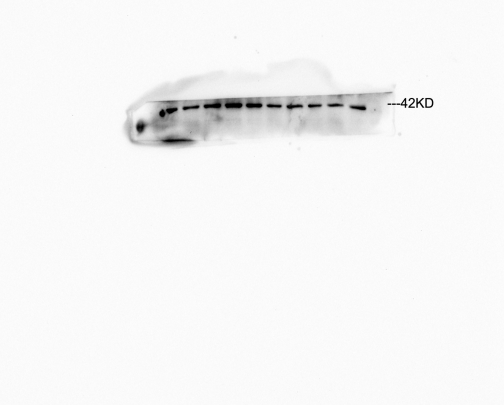

Supplement: S1 File — (DOCX) [file pgen.1011788.s027.docx]
