# Supplementary material for: Identification of hub gene associated with colorectal cancer: Integrating Mendelian randomization, transcriptome analysis and experimental verification
Source: PLoS Genet. 2025 Jul 29;21(7):e1011788. doi: 10.1371/journal.pgen.1011788 (PMC12349882; doi:10.1371/journal.pgen.1011788)
Supplement: S2 File — (DOCX) [file pgen.1011788.s028.docx]

1. **Mendelian Randomization**

R version 4.3.2 (2023-10-31 ucrt)

Platform: x86_64-w64-mingw32/x64 (64-bit)

Running under: Windows 10 x64 (build 19045)

Matrix products: default

locale:

[1] LC_COLLATE=Chinese (Simplified)_China.utf8

[2] LC_CTYPE=Chinese (Simplified)_China.utf8

[3] LC_MONETARY=Chinese (Simplified)_China.utf8

[4] LC_NUMERIC=C

[5] LC_TIME=Chinese (Simplified)_China.utf8

time zone: Asia/Shanghai

tzcode source: internal

attached base packages:

[1] stats graphics grDevices utils datasets methods base

other attached packages:

[1] TwoSampleMR_0.5.8

loaded via a namespace (and not attached):

[1] utf8_1.2.4 R6_2.5.1 tidyselect_1.2.0 magrittr_2.0.3

[5] remotes_2.4.2.1 glue_1.6.2 tibble_3.2.1 ieugwasr_0.1.5

[9] pkgconfig_2.0.3 dplyr_1.1.4 generics_0.1.3 lifecycle_1.0.4

[13] cli_3.6.2 fansi_1.0.6 vctrs_0.6.5 withr_2.5.2

[17] data.table_1.14.10 compiler_4.3.2 plyr_1.8.9 httr_1.4.7

[21] rstudioapi_0.15.0 tools_4.3.2 curl_5.2.0 pillar_1.9.0

[25] Rcpp_1.0.11 rlang_1.1.2 jsonlite_1.8.8

FIGURE:

R version 4.2.1 (2022-06-23 ucrt)

Platform: x86_64-w64-mingw32/x64 (64-bit)

Running under: Windows 10 x64 (build 19045)

Matrix products: default

locale:

[1] LC_COLLATE=Chinese (Simplified)_China.utf8

[2] LC_CTYPE=Chinese (Simplified)_China.utf8

[3] LC_MONETARY=Chinese (Simplified)_China.utf8

[4] LC_NUMERIC=C

[5] LC_TIME=Chinese (Simplified)_China.utf8

attached base packages:

[1] grid stats graphics grDevices utils datasets methods base

other attached packages:

[1] forestplot_3.1.3 abind_1.4-5 checkmate_2.3.1

loaded via a namespace (and not attached):

[1] fansi_1.0.5 utf8_1.2.4 dplyr_1.1.3 R6_2.5.1

[5] backports_1.4.1 lifecycle_1.0.4 magrittr_2.0.3 pillar_1.9.0

[9] rlang_1.1.2 cli_3.6.1 rstudioapi_0.15.0 vctrs_0.6.4

[13] generics_0.1.3 tools_4.2.1 glue_1.6.2 compiler_4.2.1

[17] pkgconfig_2.0.3 tidyselect_1.2.0 tibble_3.2.1

Code：

# Remove all objects from the current environment

rm(list = ls())

# Set stringsAsFactors to FALSE

options(stringsAsFactors = F)

# Get current working directory

getwd()

# Set working directory

setwd("D:/ZY-DAIMAHEJ/SMR/GWAS")

# Prepare SMR file format: "SNP", "A1", "A2", "freq", "b", "se", "p", "n"

library(VariantAnnotation)

library(gwasglue)

# Read VCF file

vcf <- readVcf("D:/博/孟德尔随机化 仿写/结肠癌-投稿/20250213补实验/ieu-b-4965.vcf.gz")

# Convert VCF to TwoSampleMR format

gwas <- gwasvcf_to_TwoSampleMR(vcf)

# Select specific columns

f <- gwas[, c("SNP", "effect_allele.exposure", "other_allele.exposure", "eaf.exposure",

"beta.exposure", "se.exposure", "pval.exposure", "samplesize.exposure")]

# Rename columns

colnames(f) <- c("SNP", "A1", "A2", "freq", "b", "se", "p", "n")

# Write the data to a file

write.table(f, "SMR-ieu-b-4965.txt", sep = "\t", row.names = FALSE, quote = FALSE)

# Alternative code to handle large files

library(readxl)

library(data.table)

library(tidyr)

library(dplyr)

# Read VCF file

data <- fread("ebi-a-GCST007090.vcf", data.table = F, integer64 = "numeric")

# Split the 10th column into new columns

data <- separate(data, col = 10, into = c("ES", "SE", "LP", "AF", "SNP"), sep = ":")

# Convert specific columns to numeric

data <- mutate(data, ES = as.numeric(ES), SE = as.numeric(SE), LP = as.numeric(LP), AF = as.numeric(AF))

# Calculate p-values

data <- mutate(data, pval = 10^(-LP))

# Add sample size manually

data$samplesize <- 403124

# Select relevant columns

data <- data[, c("SNP", "effect_allele", "other_allele", "AF", "ES", "SE", "pval", "samplesize")]

# Rename columns

colnames(data) <- c("SNP", "A1", "A2", "freq", "b", "se", "p", "n")

# Write the data to a file

write.table(data, "ebi-a-GCST007090.txt", sep = "\t", row.names = FALSE, quote = FALSE)

# Perform FDR correction on SMR results

data <- read.delim("1.smr", header = TRUE, stringsAsFactors = FALSE)

# Calculate FDR

fdr <- p.adjust(data$p_SMR, method = "BH")

# Create results dataframe

results <- data.frame(data, FDR = fdr)

# Structure of results

str(results)

# Filter results with FDR < 0.05

results_filtered <- subset(results, FDR < 0.05 & !is.na(FDR))

# Remove rows with NA values

results_filtered <- na.omit(results_filtered)

# Write results to CSV

write.csv(results_filtered, "eqtl-gwas.csv", row.names = FALSE)

# Calculate OR and confidence intervals

library(readr)

SMR_eqtl_all <- read.csv("eqtl-gwas.csv")

# Calculate OR and confidence intervals

SMR_eqtl_all$OR <- exp(SMR_eqtl_all$b_SMR)

SMR_eqtl_all$Lower_CI <- exp(SMR_eqtl_all$b_SMR - 1.96 * SMR_eqtl_all$se_SMR)

SMR_eqtl_all$Upper_CI <- exp(SMR_eqtl_all$b_SMR + 1.96 * SMR_eqtl_all$se_SMR)

# Format OR and confidence intervals

SMR_eqtl_all$OR_95CI <- paste(round(SMR_eqtl_all$OR, 3), "(", round(SMR_eqtl_all$Lower_CI, 3), "-", round(SMR_eqtl_all$Upper_CI, 3), ")", sep = "")

# Write results to CSV

write.csv(SMR_eqtl_all, "计算OR和置信区间.csv", row.names = FALSE)

# Convert gene IDs to symbols

matched_rows <- read.csv("计算OR和置信区间.csv")

library(clusterProfiler)

library(org.Hs.eg.db)

# Convert ENSEMBL IDs to SYMBOL

ensem <- matched_rows$probeID

genes <- bitr(ensem,

fromType = "ENSEMBL",

toType = "SYMBOL",

OrgDb = "org.Hs.eg.db",

drop = F)

# Rename columns

colnames(genes)[colnames(genes) == "ENSEMBL"] <- "probeID"

# Merge dataframes

merged_df <- merge(matched_rows, genes, by.x = "probeID", all.x = TRUE)

# Remove rows with NA in SYMBOL

merged_df <- merged_df[!is.na(merged_df$SYMBOL), ]

# Rename probeID to SYMBOL

merged_df$probeID <- merged_df$SYMBOL

# Save the merged dataframe

write.table(merged_df, file = "转换基于后文件.txt", sep = "\t", quote = F, col.names = T, row.names = F)

# Forest plot for OR and confidence intervals

SMR_eqtl_all <- read.csv("./result/过滤EUR_SMR-ieu-a-32.txt_Whole_Blood.lite.csv")

# Calculate OR and confidence intervals

SMR_eqtl_all$OR <- exp(SMR_eqtl_all$b_SMR)

SMR_eqtl_all$Lower_CI <- exp(SMR_eqtl_all$b_SMR - 1.96 * SMR_eqtl_all$se_SMR)

SMR_eqtl_all$Upper_CI <- exp(SMR_eqtl_all$b_SMR + 1.96 * SMR_eqtl_all$se_SMR)

# Format OR and confidence intervals

SMR_eqtl_all$OR_95CI <- paste(round(SMR_eqtl_all$OR, 3), "(", round(SMR_eqtl_all$Lower_CI, 3), "-", round(SMR_eqtl_all$Upper_CI, 3), ")", sep = "")

# Save to CSV

write.csv(SMR_eqtl_all, "./result/全部森林图过滤EUR_SMR-ieu-a-32.txt_Whole_Blood.lite.csv", row.names = FALSE)

# Manhatten plot and QQ plot

source("plot_SMR.r")

library(devtools)

library(AHMbook)

smrdata <- ReadSMRData("./plot/myplot.ENSG00000204410.txt")

# Create Manhatten plot

pdf(file = paste0("eqlt-AGBL2.pdf"), width = 20, height = 12)

SMRLocusPlot(data = smrdata, smr_thresh = 0.05, heidi_thresh = 0.05, plotWindow = 100, anno_selfdef = FALSE)

dev.off()

# Create QQ plot

pdf("eqtl-gwas-AGBL2.pdf", width = 5, height = 4)

SMREffectPlot(data = smrdata)

dev.off()

# Additional plots

SMRLocusPlot(data = smrdata, smr_thresh = 8.4e-6, heidi_thresh = 0.05, plotWindow = 1000)

SMRLocusPlot(data = smrdata, smr_thresh = 8.4e-8, heidi_thresh = 0.05, plotWindow = 1000)

SMRLocusPlot(data = smrdata, smr_thresh = 8.4e-6, heidi_thresh = 0.05, plotWindow = 1000, smr_thresh_plot = 1e-1)

SMRLocusPlot(data = smrdata, smr_thresh = 8.4e-6, heidi_thresh = 0.05, plotWindow = 1000, probeNEARBY = c("ILMN_1724700", "ILMN_does_not_exist"))

# Part IX: Decode all pqtl requests------------------------------------------------------------------------------------------

rm(list = ls())

options(stringsAsFactors = F)

# Set working directory

afdir <- paste0(getwd(), "/pqtl")

# Load required libraries

library(TwoSampleMR)

library(readxl)

library(gridExtra)

library(forestplot)

library(scales)

library(tidyverse)

# Read Excel files

# pqtl_csf <- read_excel("genetic_instrument_exposure_CSF_protein.xlsx")

pqtl_plasma <- read_excel("1553_pQTL_deCODE_clumped.xlsx")

# Extract outcome data from the OpenGWAS project

snp_MS <- extract_outcome_data(

snps = unique(c(pqtl_plasma$SNP)),

outcomes = "ukb-b-17670"

)

writexl::write_xlsx(snp_MS, "snp_MS.xlsx")

# Read VCF file locally

library(VariantAnnotation)

library(gwasglue)

vcf <- readVcf("ukb-b-17670.vcf.gz")

exp_dat <- gwasvcf_to_TwoSampleMR(vcf)

exp_dat$id.exposure = "Multiple Sclerosis"

# Format the exposure data

f <- exp_dat[, c("SNP", "effect_allele.exposure", "other_allele.exposure", "eaf.exposure",

"beta.exposure", "se.exposure", "pval.exposure", "samplesize.exposure",

"chr.exposure", "pos.exposure"

)]

# Normalize exposure format

out_dat <- TwoSampleMR::format_data(f, type = "outcome", snp_col = "SNP",

beta_col = "beta.exposure", se_col = "se.exposure",

eaf_col = "eaf.exposure",

effect_allele_col = "effect_allele.exposure",

other_allele_col = "other_allele.exposure",

pval_col = "pval.exposure",

samplesize_col = "samplesize.exposure",

chr_col = "chr.exposure", pos_col = "pos.exposure"

)

out_dat$id.outcome = "Multiple Sclerosis"

snp_MS <- out_dat

# Harmonize plasma and MS data

harmonised_plasma_MS <- harmonise_data(pqtl_plasma, snp_MS)

# Define a modified MR function

mr_modified <- function(dat = harmonised_csf_MS, prop_var_explained = T) {

mr_res <- mr(dat)

pve <- dat %>%

dplyr::select(id.exposure, beta.exposure, se.exposure, samplesize.exposure) %>%

dplyr::group_by(id.exposure) %>%

dplyr::summarise(pve = sum((beta.exposure^2)/(beta.exposure^2 + samplesize.exposure * se.exposure^2)))

if (prop_var_explained) {

mr_res <- mr_res %>%

dplyr::left_join(pve, by = "id.exposure")

}

return(mr_res)

}

# Perform MR analysis for plasma and MS

mr_plasma_MS <- mr_modified(harmonised_plasma_MS, prop_var_explained = T)

# Heterogeneity and pleiotropy tests

heterogeneity_plasma_MS <- mr_heterogeneity(harmonised_plasma_MS)

pleiotropy_plasma_MS <- mr_pleiotropy_test(harmonised_plasma_MS)

# Save results to CSV

write.csv(heterogeneity_plasma_MS, "heterogeneity_plasma_MS.csv")

write.csv(pleiotropy_plasma_MS, "pleiotropy_plasma_MS.csv")

# Apply Bonferroni correction

table2_plasma <- mr_plasma_MS %>%

dplyr::filter(pval < 0.05 / 1553,

method %in% c("Wald ratio", "Inverse variance weighted"))

# Apply FDR correction

fdr <- p.adjust(mr_plasma_MS$pval, method = "BH")

results <- data.frame(mr_plasma_MS, FDR = fdr)

results_filtered <- subset(results, FDR < 0.05 & !is.na(FDR))

results_filtered <- na.omit(results_filtered)

write.csv(results_filtered, "PQTL-FDR矫正.csv", row.names = FALSE)

# Format results for publication

table2_plasma$OR <- exp(table2_plasma$b)

table2_plasma$Lower_CI <- exp(table2_plasma$b - 1.96 * table2_plasma$se)

table2_plasma$Upper_CI <- exp(table2_plasma$b + 1.96 * table2_plasma$se)

table2_plasma$OR_95CI <- paste(round(table2_plasma$OR, 3), "(", round(table2_plasma$Lower_CI, 3),

"-", round(table2_plasma$Upper_CI, 3), ")", sep = "")

write_csv(table2_plasma, file = "peql.csv")

# Sensitivity analysis

table1_plasma <- mr_plasma_MS %>%

dplyr::filter(pval < 0.05 / 4441,

method %in% c("Wald ratio", "Inverse variance weighted")) %>%

left_join(pqtl_plasma, by = "exposure")

table1 <- table1_plasma %>%

generate_odds_ratios() %>%

mutate(`OR (95% CI)` = sprintf("%.2f (%.2f, %.2f)", or, or_lci95, or_uci95),

`P value` = scales::scientific(pval),

`PVE` = paste0(sprintf("%.2f", 100 * pve), "%"),

`F statistics` = sprintf("%.2f", F_statistics)) %>%

dplyr::select(Tissue, Protein = exposure, `UniProt ID` = UniProt,

SNP, `Effect allele` = effect_allele.exposure,

`OR (95% CI)`, `P value`,

PVE, `F statistics`, Author)

writexl::write_xlsx(table1, "table1.xlsx")

save(harmonised_plasma_MS, table1, table2_plasma, mr_plasma_MS, pqtl_plasma, snp_MS, file = "pqtl数据.rdata")

writexl::write_xlsx(table2_plasma, "pqtl阳性.xlsx")

# Volcano plot for main MR findings

volcano_plot <- function(.data,

number_comparasion = 1,

title = "(A)",

col_beta = "b",

col_size = "pve",

col_label = "exposure",

legend.position = "none") {

p_thershold <- 0.05 / number_comparasion

p <- .data %>%

rename_with(~ case_when(

.x == col_beta ~ "beta",

.x == col_size ~ "size",

.x == col_label ~ "label",

TRUE ~ .x

)) %>%

mutate(x = beta,

y = -log10(pval),

label = ifelse(pval < p_thershold, label, NA)) %>%

ggplot(aes(x = x, y = y)) +

geom_point(aes(size = size), alpha = 0.5, color = "#B80000") +

geom_vline(xintercept = 0, linetype = 2) +

geom_hline(yintercept = -log10(p_thershold), linetype = 2) +

theme_classic() +

theme(panel.grid = element_blank(),

legend.title = element_text(size = 6.5),

legend.text = element_text(size = 6.5),

legend.position = legend.position) +

labs(x = "ln(OR)",

y = parse(text = "-log[10]*(italic(P)-value)"),

title = title) +

scale_size(name = "PVE",

breaks = c(0.2 * 1:3)) +

ggrepel::geom_label_repel(aes(label = label), size = 3)

plot(p)

}

library(ensembldb)

pdf(file = paste0(afdir, "/阳性火山图.pdf"), width = 10, height = 7)

gridExtra::grid.arrange(

dplyr::filter(mr_plasma_MS, method %in% c("Wald ratio", "Inverse variance weighted")) %>%

volcano_plot(number_comparasion = 4441,

title = "A Plasma"),

ncol = 1, nrow = 1) +

scale_size(name = "PVE", breaks = c(0.2 * 1:3)) +

ggrepel::geom_label_repel(aes(label = label), size = 3)

dev.off()

# Sensitivity analysis for PPI

table_ext_ukb <- table_ext %>%

filter(id.outcome == "MS_UKB") %>%

generate_table_for_forestplot() %>%

dplyr::select(exposure, tissue, source, Odds:High, pval, ci) %>%

add_title_for_table()

table_ext_finn <- table_ext %>%

filter(id.outcome == "MS_Finn") %>%

generate_table_for_forestplot() %>%

dplyr::select(exposure, tissue, source, Odds:High, pval, ci) %>%

add_title_for_table()

# Generate forest plots

forestplot(labeltext = as.matrix(table_ext_ukb[, c("exposure", "tissue", "source", "ci", "pval")]),

mean = table_ext_ukb$Odds, lower = table_ext_ukb$Low, upper = table_ext_ukb$High,

align = "l",

is.summary = c(T, rep(c(F, F, F), 12)),

graph.pos = 4,

hrzl_lines = list("2" = gpar(lty = 1, col = "black")),

xlab = paste0(c("Lower ← → Higher", paste0(rep("", 0), collapse = "")), collapse = ""),

zero = 1,

graphwidth = unit(5, 'cm'),

colgap = unit(10, 'mm'),

lineheight = unit(6, 'mm'),

col = fpColors(box = 'black', lines = 'black', zero = 'lightgray'),

txt_gp = fpTxtGp(label = gpar(cex = 0.85), ticks = gpar(cex = 0.85), xlab = gpar(cex = 0.85)),

xlog = F,

xticks = c(0.25, 1, 4),

clip = c(0.25, 4),

xticks.digits = 2,

lwd.xaxis = 1,

lwd.zero = 1,

lwd.ci = 1,

lty.ci = 1,

ci.vertices = F,

boxsize = 0.15,

mar = unit(rep(0, times = 4), "mm"),

new_page = T,

fn.ci_norm = fpDrawNormalCI)

forestplot(labeltext = as.matrix(table_ext_finn[, c("exposure", "tissue", "source", "ci", "pval")]),

mean = table_ext_finn$Odds, lower = table_ext_finn$Low, upper = table_ext_finn$High,

align = "l",

is.summary = c(T, rep(c(F, F, F), 12)),

graph.pos = 4,

hrzl_lines = list("2" = gpar(lty = 1, col = "black")),

xlab = paste0(c("Lower ← → Higher", paste0(rep("", 0), collapse = "")), collapse = ""),

zero = 1,

graphwidth = unit(5, 'cm'),

colgap = unit(10, 'mm'),

lineheight = unit(6, 'mm'),

col = fpColors(box = 'black', lines = 'black', zero = 'lightgray'),

txt_gp = fpTxtGp(label = gpar(cex = 0.85), ticks = gpar(cex = 0.85), xlab = gpar(cex = 0.85)),

xlog = F,

xticks = c(0.25, 1, 4),

clip = c(0.25, 4),

xticks.digits = 2,

lwd.xaxis = 1,

lwd.zero = 1,

lwd.ci = 1,

lty.ci = 1,

ci.vertices = F,

boxsize = 0.15,

mar = unit(rep(0, times = 4), "mm"),

new_page = T,

fn.ci_norm = fpDrawNormalCI)

dev.off()

# Colocalization analysis

rm(list = ls())

options(stringsAsFactors = F)

afdir <- paste0(getwd(), "/共定位")

# Function to process Excel files

process_excel <- function(file) {

library(readxl)

data <- read_excel(file)

library(tidyr)

data <- separate(data, col = 10, into = c("ES", "SE", "LP", "AF", "SS", "SNP"), sep = ":")

library(dplyr)

data <- mutate(data, ES = as.numeric(ES), SE = as.numeric(SE), LP = as.numeric(LP), AF = as.numeric(AF), SS = as.numeric(SS))

data <- mutate(data, pval = 10^(-LP))

colnames(data) <- c("chr", "pos", "SNP", "effect_allele", "other_allele",

"QUAL", "FILTER", "INFO", "FORMAT",

"beta", "se", "LP", "maf", "samplesize", "id", "pval"

)

data$beta[is.infinite(data$beta)] <- NA

data$se[is.infinite(data$se)] <- NA

data <- na.omit(data)

duplicated_rows <- duplicated(data$SNP)

data <- data[!duplicated_rows, ]

new_name <- paste0(gsub("\\.xlsx$", "", file))

assign(new_name, data, envir = .GlobalEnv)

}

# List of Excel files to process

files <- c("STX7.xlsx", "RPL34.xlsx", "CD27.xlsx", "XCL2.xlsx", "CTSW.xlsx",

"EIF1.xlsx", "C2orf88.xlsx", "HLA_DRB5.xlsx", "HLA_C.xlsx",

"HLA_G.xlsx", "HLA_A.xlsx", "LTB.xlsx", "RPS18.xlsx",

"HLA_B.xlsx", "HLA_DQA2.xlsx", "PSMB9.xlsx")

lapply(files, process_excel)

# Function to extract outcome data

extract_coloc_outcome_dat <- function(coloc_exposure_dat,

outcome_id = "ukb-b-17670") {

coloc_outcome_dat <-

extract_outcome_data(snps = ,

outcomes = "ukb-b-17670",

proxies = F) %>%

mutate(chr.outcome = as.numeric(chr),

pos.outcome = as.numeric(pos),

outcome = "outcome",

id.outcome = "ukb-b-17670")

return(coloc_outcome_dat)

}

coloc_MS_dat <- extract_outcome_data(snps = c(CMPK1$SNP,

HLA_C$SNP,

LTB$SNP

),

outcomes = "finn-b-G6_MS",

proxies = F) %>%

mutate(chr.outcome = as.numeric(chr),

pos.outcome = as.numeric(pos),

outcome = "MultipleSclerosis",

id.outcome = "finn-b-G6_MS")

library(VariantAnnotation)

library(gwasglue)

vcf <- readVcf("ukb-b-17670.vcf.gz")

exp_dat <- gwasvcf_to_TwoSampleMR(vcf)

exp_dat$id.exposure = "Multiple Sclerosis"

f <- exp_dat[, c("SNP", "effect_allele.exposure", "other_allele.exposure", "eaf.exposure",

"beta.exposure", "se.exposure", "pval.exposure", "samplesize.exposure",

"chr.exposure", "pos.exposure"

)]

# Normalize outcome data

out_dat <- TwoSampleMR::format_data(f, type = "outcome", snp_col = "SNP",

beta_col = "beta.exposure", se_col = "se.exposure",

eaf_col = "eaf.exposure",

effect_allele_col = "effect_allele.exposure",

other_allele_col = "other_allele.exposure",

pval_col = "pval.exposure",

samplesize_col = "samplesize.exposure",

chr_col = "chr.exposure", pos_col = "pos.exposure"

)

out_dat$id.outcome = "Multiple Sclerosis"

coloc_MS_dat <- out_dat

duplicated_rows <- duplicated(coloc_MS_dat$SNP)

coloc_MS_dat <- coloc_MS_dat[!duplicated_rows, ]

save(coloc_MS_dat, file = '共定位结局数据.rdata')

load("共定位结局数据.rdata")

# Modify the coloc_test function

coloc_test <- function(exposure_dat,

outcome_dat,

type_exposure = "quant",

col_pvalues_exposure = "pval",

col_N_exposure = "samplesize",

col_MAF_exposure = "maf",

col_beta_exposure = "beta",

col_se_exposure = "se",

col_snp_exposure = "SNP",

sd_exposure = NA,

type_outcome = "cc",

col_pvalues_outcome = "pval.outcome",

col_N_outcome = "samplesize.outcome",

col_MAF_outcome = "eaf.outcome",

col_beta_outcome = "beta.outcome",

col_se_outcome = "se.outcome",

col_snp_outcome = "SNP",

prevalence_outcome = 0.0036) {

cols_exposure <- c(col_pvalues_exposure,

col_N_exposure,

col_MAF_exposure,

col_beta_exposure,

col_se_exposure,

col_snp_exposure)

cols_exposure <- cols_exposure[!is.na(cols_exposure)]

cols_outcome <- c(col_pvalues_outcome,

col_N_outcome,

col_MAF_outcome,

col_beta_outcome,

col_se_outcome,

col_snp_outcome)

cols_outcome <- cols_outcome[!is.na(cols_outcome)]

stopifnot(all(cols_exposure %in% names(exposure_dat)))

stopifnot(all(cols_outcome %in% names(outcome_dat)))

snp_overlap <- intersect(exposure_dat[[col_snp_exposure]],

outcome_dat[[col_snp_outcome]])

snp_overlap <- unique(snp_overlap)

exposure_dat <- exposure_dat[exposure_dat[[col_snp_exposure]] %in% snp_overlap, ]

outcome_dat <- outcome_dat[outcome_dat[[col_snp_outcome]] %in% snp_overlap, ]

exposure_dat <- exposure_dat[order(exposure_dat[[col_snp_exposure]]), ]

outcome_dat <- outcome_dat[order(outcome_dat[[col_snp_outcome]]), ]

exposure_list <- list()

outcome_list <- list()

for (i in 1:8) {

list_element <- c("pvalues", "N", "MAF",

"beta", "varbeta", "snp",

"type", "sdY")[i]

col_element <- c(col_pvalues_exposure, col_N_exposure, col_MAF_exposure,

col_beta_exposure, col_se_exposure, col_snp_exposure,

type_exposure, sd_exposure)[i]

if (!is.na(col_element)) {

if (list_element %in% c("type", "sdY")) {

if (list_element == "sdY")

col_element <- as.numeric(col_element)

exposure_list[[list_element]] <- col_element

} else {

if (list_element == "varbeta") {

exposure_list[[list_element]] <- exposure_dat[[col_element]] * exposure_dat[[col_element]]

} else {

exposure_list[[list_element]] <- exposure_dat[[col_element]]

}

}

}

}

for (j in 1:8) {

list_element <- c("pvalues", "N", "MAF",

"beta", "varbeta", "snp",

"type", "s")[j]

col_element <- c(col_pvalues_outcome, col_N_outcome, col_MAF_outcome,

col_beta_outcome, col_se_outcome, col_snp_outcome,

type_outcome, prevalence_outcome)[j]

if (!is.na(col_element)) {

if (list_element %in% c("type", "s")) {

if (list_element == "s")

col_element <- as.numeric(col_element)

outcome_list[[list_element]] <- col_element

} else {

if (list_element == "varbeta") {

outcome_list[[list_element]] <- outcome_dat[[col_element]] * outcome_dat[[col_element]]

} else {

outcome_list[[list_element]] <- outcome_dat[[col_element]]

}

}

}

}

coloc::coloc.abf(exposure_list, outcome_list, p1 = 1e-04, p2 = 1e-04, p12 = 1e-05)

}

library(coloc)

# Single SNP colocalization

library(tibble)

coloc_proteins_MS <- coloc_test(exposure_dat = STX7,

outcome_dat = coloc_MS_dat)$summary %>%

enframe() %>% mutate(exposure = "STX7") %>%

bind_rows(

coloc_test(exposure_dat = RPL34,

outcome_dat = coloc_MS_dat)$summary %>%

enframe() %>% mutate(exposure = "RPL34")) %>%

bind_rows(

coloc_test(exposure_dat = CD27,

outcome_dat = coloc_MS_dat)$summary %>%

enframe() %>% mutate(exposure = "CD27")) %>%

bind_rows(

coloc_test(exposure_dat = XCL2,

outcome_dat = coloc_MS_dat)$summary %>%

enframe() %>% mutate(exposure = "XCL2")) %>%

bind_rows(

coloc_test(exposure_dat = CTSW,

outcome_dat = coloc_MS_dat)$summary %>%

enframe() %>% mutate(exposure = "CTSW")) %>%

bind_rows(

coloc_test(exposure_dat = EIF1,

outcome_dat = coloc_MS_dat)$summary %>%

enframe() %>% mutate(exposure = "EIF1")) %>%

bind_rows(

coloc_test(exposure_dat = C2orf88,

outcome_dat = coloc_MS_dat)$summary %>%

enframe() %>% mutate(exposure = "C2orf88")) %>%

bind_rows(

coloc_test(exposure_dat = HLA_DRB5,

outcome_dat = coloc_MS_dat)$summary %>%

enframe() %>% mutate(exposure = "HLA_DRB5")) %>%

bind_rows(

coloc_test(exposure_dat = HLA_C,

outcome_dat = coloc_MS_dat)$summary %>%

enframe() %>% mutate(exposure = "HLA_C")) %>%

bind_rows(

coloc_test(exposure_dat = HLA_G,

outcome_dat = coloc_MS_dat)$summary %>%

enframe() %>% mutate(exposure = "HLA_G")) %>%

bind_rows(

coloc_test(exposure_dat = HLA_A,

outcome_dat = coloc_MS_dat)$summary %>%

enframe() %>% mutate(exposure = "HLA_A")) %>%

bind_rows(

coloc_test(exposure_dat = LTB,

outcome_dat = coloc_MS_dat)$summary %>%

enframe() %>% mutate(exposure = "LTB")) %>%

bind_rows(

coloc_test(exposure_dat = RPS18,

outcome_dat = coloc_MS_dat)$summary %>%

enframe() %>% mutate(exposure = "RPS18")) %>%

bind_rows(

coloc_test(exposure_dat = HLA_B,

outcome_dat = coloc_MS_dat)$summary %>%

enframe() %>% mutate(exposure = "HLA_B")) %>%

bind_rows(

coloc_test(exposure_dat = HLA_DQA2,

outcome_dat = coloc_MS_dat)$summary %>%

enframe() %>% mutate(exposure = "HLA_DQA2")) %>%

bind_rows(

coloc_test(exposure_dat = PSMB9,

outcome_dat = coloc_MS_dat)$summary %>%

enframe() %>% mutate(exposure = "PSMB9"))

coloc_proteins_MS <- coloc_proteins_MS %>%

pivot_wider(names_from = "name", values_from = "value") %>%

mutate(exposure = factor(exposure,

levels = c("STX7",

"RPL34",

"CD27",

"XCL2",

"CTSW",

"EIF1",

"C2orf88",

"HLA_DRB5",

"HLA_C",

"HLA_G",

"HLA_A",

"LTB",

"RPS18",

"HLA_B",

"HLA_DQA2",

"PSMB9"

))) %>%

arrange(exposure) %>%

mutate(PP.H3.abf = sprintf("%.3f", PP.H3.abf),

PP.H4.abf = sprintf("%.3f", PP.H4.abf))

write.csv(coloc_proteins_MS, "coloc_proteins_MS2.csv")

coloc_plot <- function(exposure_dat = coloc_SLAF7_dat,

exposure = "SLAF7",

col_snp_exposure = "SNP",

col_pvalues_exposure = "pval",

outcome_dat = coloc_MS_dat,

outcome = "MS",

col_snp_outcome = "SNP",

col_pvalues_outcome = "pval.outcome",

path = getwd(),

combine = FALSE,

legend = FALSE) {

library(locuscomparer)

snp_overlap <- intersect(exposure_dat[[col_snp_exposure]],

outcome_dat[[col_snp_outcome]])

snp_overlap <- unique(snp_overlap)

exposure_dat <- exposure_dat[exposure_dat[[col_snp_exposure]] %in% snp_overlap, ]

outcome_dat <- outcome_dat[outcome_dat[[col_snp_outcome]] %in% snp_overlap, ]

exposure_dat <- exposure_dat[order(exposure_dat[[col_snp_exposure]]), ]

outcome_dat <- outcome_dat[order(outcome_dat[[col_snp_outcome]]), ]

exposure_dat <- data.frame(

rsid = exposure_dat[[col_snp_exposure]],

pval = exposure_dat[[col_pvalues_exposure]]

)

outcome_dat <- data.frame(

rsid = outcome_dat[[col_snp_outcome]],

pval = outcome_dat[[col_pvalues_outcome]]

)

write.table(exposure_dat, paste0(path, "/exposure_test.tsv"), sep = "\t", row.names = F, quote = F)

write.table(outcome_dat, paste0(path, "/outcome_test.tsv"), sep = "\t", row.names = F, quote = F)

p <- locuscompare(in_fn1 = paste0(path, "/outcome_test.tsv"),

in_fn2 = paste0(path, "/exposure_test.tsv"),

title1 = paste0(outcome, " GWAS"),

title2 = paste0(exposure, " eQTL"),

combine = combine,

legend = legend)

p

}

save(coloc_MS_dat, coloc_proteins_MS, file = '共定位结局数据.rdata')

pdf(file = paste0(afdir, "/共定位.pdf"), width = 15, height = 12)

gridExtra::grid.arrange(

coloc_plot(exposure_dat = CD27, exposure = "CD27", combine = F, legend = T)$locuscompare + ggtitle("(A)"),

coloc_plot(exposure_dat = AHI1, exposure = "AHI1", combine = F)$locuscompare + ggtitle("(B)"),

coloc_plot(exposure_dat = SLC39A13, exposure = "SLC39A13", combine = F)$locuscompare + ggtitle("(C)"),

coloc_plot(exposure_dat = PSMC3, exposure = "PSMC3", combine = F)$locuscompare + ggtitle("(D)"),

coloc_plot(exposure_dat = AGBL2, exposure = "AGBL2", combine = F)$locuscompare + ggtitle("(E)"),

ncol = 3

)

dev.off()

**2. Immune checkpoint correlation analysis**

R version 4.2.1 (2022-06-23 ucrt)

Platform: x86_64-w64-mingw32/x64 (64-bit)

Running under: Windows 10 x64 (build 19045)

Matrix products: default

locale:

[1] LC_COLLATE=Chinese (Simplified)_China.utf8

[2] LC_CTYPE=Chinese (Simplified)_China.utf8

[3] LC_MONETARY=Chinese (Simplified)_China.utf8

[4] LC_NUMERIC=C

[5] LC_TIME=Chinese (Simplified)_China.utf8

attached base packages:

[1] parallel tcltk stats graphics grDevices utils datasets methods

[9] base

other attached packages:

[1] corrplot_0.92 ggExtra_0.10.0 vioplot_0.4.0

[4] zoo_1.8-12 sm_2.2-5.7.1 data.table_1.14.8

[7] doParallel_1.0.17 iterators_1.0.14 foreach_1.5.2

[10] preprocessCore_1.62.1 e1071_1.7-13 ridge_3.3

[13] car_3.1-2 carData_3.0-5 future.apply_1.11.0

[16] future_1.33.0 ggpubr_0.6.0 limma_3.54.2

[19] reshape2_1.4.4 ggtext_0.1.2 scales_1.3.0

[22] ggsci_3.0.0 lubridate_1.9.3 forcats_1.0.0

[25] stringr_1.5.0 dplyr_1.1.3 purrr_1.0.2

[28] readr_2.1.4 tidyr_1.3.0 tibble_3.2.1

[31] ggplot2_3.4.4 tidyverse_2.0.0

loaded via a namespace (and not attached):

[1] fs_1.6.3 usethis_2.2.2 devtools_2.4.5 profvis_0.3.8

[5] tools_4.2.1 backports_1.4.1 utf8_1.2.4 R6_2.5.1

[9] colorspace_2.1-0 urlchecker_1.0.1 withr_2.5.2 prettyunits_1.2.0

[13] tidyselect_1.2.0 processx_3.8.2 compiler_4.2.1 cli_3.6.1

[17] xml2_1.3.5 labeling_0.4.3 callr_3.7.3 proxy_0.4-27

[21] digest_0.6.33 pkgconfig_2.0.3 htmltools_0.5.7 sessioninfo_1.2.2

[25] parallelly_1.36.0 fastmap_1.1.1 htmlwidgets_1.6.3 rlang_1.1.2

[29] rstudioapi_0.15.0 shiny_1.7.5.1 farver_2.1.1 generics_0.1.3

[33] magrittr_2.0.3 Rcpp_1.0.11 munsell_0.5.0 fansi_1.0.5

[37] abind_1.4-5 lifecycle_1.0.4 stringi_1.7.12 pkgbuild_1.4.2

[41] plyr_1.8.9 grid_4.2.1 listenv_0.9.0 promises_1.2.1

[45] crayon_1.5.2 miniUI_0.1.1.1 lattice_0.20-45 gridtext_0.1.5

[49] hms_1.1.3 ps_1.7.5 pillar_1.9.0 ggsignif_0.6.4

[53] pkgload_1.3.3 codetools_0.2-19 glue_1.6.2 remotes_2.4.2.1

[57] vctrs_0.6.4 tzdb_0.4.0 httpuv_1.6.12 gtable_0.3.4

[61] cachem_1.0.8 mime_0.12 xtable_1.8-4 broom_1.0.5

[65] rstatix_0.7.2 later_1.3.1 class_7.3-22 memoise_2.0.1

[69] timechange_0.2.0 globals_0.16.2 ellipsis_0.3.2

Code：

library(limma)

library(reshape2)

library(ggplot2)

library(ggpubr)

library(corrplot)

pFilter=0.001

co=0.4

geneName="TPX2"

expFile="combined_RNAseq_TPM.txt"

geneFile="gene.txt"

rt=read.table(expFile, header=T, sep="\t", check.names=F)

data=avereps(rt)

gene=read.table(geneFile, header=F, sep="\t", check.names=F)

sameGene=intersect(row.names(data), as.vector(gene[,1]))

data=t(data[c(geneName, sameGene),])

data=log2(data+1)

group=sapply(strsplit(row.names(data),"\\-"),"[",4)

group=sapply(strsplit(group,""),"[",1)

group=gsub("2","1",group)

data=data[group==0,]

row.names(data)=gsub("(.*?)\\-(.*?)\\-(.*?)\\-.*", "\\1\\-\\2\\-\\3", row.names(data))

data=t(avereps(data))

x=as.numeric(data[geneName,])

outTab=data.frame()

for(i in sameGene){

if(i==geneName){next}

y=as.numeric(data[i,])

corT=cor.test(x, y, method = 'pearson')

cor=corT$estimate

pvalue=corT$p.value

if(pvalue<pFilter&cor>co){

outTab=rbind(outTab, cbind(Query=geneName, Gene=i, cor, pvalue))

}

}

write.table(file="corResult.txt", outTab, sep="\t", quote=F, row.names=F)

data=t(data[c(geneName, as.vector(outTab[,2])),])

M=cor(data)

pdf(file="cor.pdf",width=8,height=8)

corrplot(M,

order="original",

method = "color",

number.cex = 0.8,

addCoef.col = "black",

diag = TRUE,

tl.col="black",

col=colorRampPalette(c("blue", "white", "red"))(50))

dev.off()

**3. Immune infiltration analysis**

R version 4.2.1 (2022-06-23 ucrt)

Platform: x86_64-w64-mingw32/x64 (64-bit)

Running under: Windows 10 x64 (build 19045)

Matrix products: default

locale:

[1] LC_COLLATE=Chinese (Simplified)_China.utf8

[2] LC_CTYPE=Chinese (Simplified)_China.utf8

[3] LC_MONETARY=Chinese (Simplified)_China.utf8

[4] LC_NUMERIC=C

[5] LC_TIME=Chinese (Simplified)_China.utf8

attached base packages:

[1] parallel tcltk stats graphics

[5] grDevices utils datasets methods

[9] base

other attached packages:

[1] immunedeconv_2.1.0

[2] data.table_1.14.8

[3] doParallel_1.0.17

[4] iterators_1.0.14

[5] foreach_1.5.2

[6] preprocessCore_1.62.1

[7] e1071_1.7-13

[8] ridge_3.3

[9] car_3.1-2

[10] carData_3.0-5

[11] future.apply_1.11.0

[12] future_1.33.0

[13] ggpubr_0.6.0

[14] limma_3.54.2

[15] reshape2_1.4.4

[16] ggtext_0.1.2

[17] scales_1.3.0

[18] ggsci_3.0.0

[19] lubridate_1.9.3

[20] forcats_1.0.0

[21] stringr_1.5.0

[22] dplyr_1.1.3

[23] purrr_1.0.2

[24] readr_2.1.4

[25] tidyr_1.3.0

[26] tibble_3.2.1

[27] ggplot2_3.4.4

[28] tidyverse_2.0.0

[29] job_0.3.0

[30] EPIC_1.1.7

loaded via a namespace (and not attached):

[1] utf8_1.2.4

[2] tidyselect_1.2.0

[3] RSQLite_2.3.4

[4] AnnotationDbi_1.60.2

[5] htmlwidgets_1.6.4

[6] grid_4.2.1

[7] BiocParallel_1.32.6

[8] lpSolve_5.6.20

[9] devtools_2.4.5

[10] ScaledMatrix_1.8.1

[11] munsell_0.5.0

[12] codetools_0.2-19

[13] S4Arrays_1.0.4

[14] miniUI_0.1.1.1

[15] withr_2.5.2

[16] colorspace_2.1-0

[17] limSolve_1.5.7

[18] Biobase_2.58.0

[19] filelock_1.0.3

[20] mMCPcounter_1.1.0

[21] rstudioapi_0.15.0

[22] stats4_4.2.1

[23] SingleCellExperiment_1.22.0

[24] ggsignif_0.6.4

[25] listenv_0.9.0

[26] labeling_0.4.3

[27] MatrixGenerics_1.14.0

[28] GenomeInfoDbData_1.2.9

[29] farver_2.1.1

[30] bit64_4.0.5

[31] rhdf5_2.44.0

[32] rprojroot_2.0.4

[33] parallelly_1.36.0

[34] vctrs_0.6.4

[35] generics_0.1.3

[36] xfun_0.41

[37] timechange_0.2.0

[38] BiocFileCache_2.6.1

[39] markdown_1.11

[40] R6_2.5.1

[41] GenomeInfoDb_1.34.9

[42] rsvd_1.0.5

[43] locfit_1.5-9.8

[44] bitops_1.0-7

[45] rhdf5filters_1.12.1

[46] cachem_1.0.8

[47] DelayedArray_0.24.0

[48] promises_1.2.1

[49] vroom_1.6.5

[50] gtable_0.3.4

[51] beachmat_2.16.0

[52] globals_0.16.2

[53] sva_3.48.0

[54] processx_3.8.2

[55] rlang_1.1.2

[56] genefilter_1.82.1

[57] splines_4.2.1

[58] rstatix_0.7.2

[59] broom_1.0.5

[60] BiocManager_1.30.22

[61] abind_1.4-5

[62] backports_1.4.1

[63] httpuv_1.6.12

[64] gridtext_0.1.5

[65] tools_4.2.1

[66] usethis_2.2.2

[67] ellipsis_0.3.2

[68] xCell_1.1.0

[69] proxy_0.4-27

[70] BiocGenerics_0.44.0

[71] sessioninfo_1.2.2

[72] testit_0.13

[73] Rcpp_1.0.11

[74] plyr_1.8.9

[75] sparseMatrixStats_1.12.0

[76] progress_1.2.3

[77] zlibbioc_1.44.0

[78] RCurl_1.98-1.13

[79] ps_1.7.5

[80] prettyunits_1.2.0

[81] urlchecker_1.0.1

[82] S4Vectors_0.36.2

[83] SummarizedExperiment_1.28.0

[84] fs_1.6.3

[85] magrittr_2.0.3

[86] matrixStats_1.2.0

[87] pkgload_1.3.3

[88] hms_1.1.3

[89] mime_0.12

[90] GSVA_1.48.0

[91] xtable_1.8-4

[92] XML_3.99-0.16

[93] readxl_1.4.3

[94] IRanges_2.32.0

[95] compiler_4.2.1

[96] biomaRt_2.54.1

[97] crayon_1.5.2

[98] htmltools_0.5.7

[99] mgcv_1.9-0

[100] later_1.3.1

[101] tzdb_0.4.0

[102] ConsensusTME_0.0.1.9000

[103] DBI_1.1.3

[104] dbplyr_2.4.0

[105] MASS_7.3-60

[106] rappdirs_0.3.3

[107] data.tree_1.1.0

[108] Matrix_1.6-4

[109] cli_3.6.1

[110] quadprog_1.5-8

[111] GenomicRanges_1.50.2

[112] pkgconfig_2.0.3

[113] xml2_1.3.5

[114] ComICS_1.0.4

[115] annotate_1.76.0

[116] XVector_0.38.0

[117] callr_3.7.3

[118] digest_0.6.33

[119] pracma_2.4.2

[120] quantiseqr_1.8.0

[121] graph_1.78.0

[122] Biostrings_2.66.0

[123] cellranger_1.1.0

[124] edgeR_3.40.2

[125] DelayedMatrixStats_1.22.0

[126] GSEABase_1.62.0

[127] curl_5.1.0

[128] commonmark_1.9.0

[129] shiny_1.8.0

[130] lifecycle_1.0.4

[131] nlme_3.1-163

[132] Rhdf5lib_1.22.0

[133] desc_1.4.3

[134] fansi_1.0.5

[135] pillar_1.9.0

[136] lattice_0.20-45

[137] KEGGREST_1.38.0

[138] fastmap_1.1.1

[139] httr_1.4.7

[140] pkgbuild_1.4.2

[141] survival_3.5-7

[142] glue_1.6.2

[143] remotes_2.4.2.1

[144] png_0.1-8

[145] bit_4.0.5

[146] class_7.3-22

[147] stringi_1.7.12

[148] profvis_0.3.8

[149] HDF5Array_1.28.1

[150] blob_1.2.4

[151] BiocSingular_1.16.0

[152] memoise_2.0.1

[153] irlba_2.3.5.1

Code：

library(immunedeconv)

library(job)

library(tcltk)

library(tidyverse)

library(ggsci)

library(scales)

library(ggtext)

library(reshape2)

library(limma)

library(parallel)

library(ggplot2)

library(ggpubr)

library(future.apply)

library(car)

library(ridge)

library(e1071)

library(preprocessCore)

library(foreach)

library(doParallel)

library(data.table)

randomColor <- function() {

paste0("#",paste0(sample(c(0:9, letters[1:6]), 6, replace = TRUE),collapse = ""))

}

randomColors <- replicate(200,randomColor())

setwd("D:")

exprMatrix <- read.table("TCGA_all_TPM.txt",header=TRUE,sep = "\t",as.is = T,row.names = 1)

exprMatrix= exprMatrix%>% dplyr::select(str_which(colnames(.), ".01"))%>%as.data.frame()

cancer="GBM"

brca_vector <- rep(cancer, ncol(exprMatrix))

#estimate

exp=exprMatrix

res_estimate <- deconvolute(exp, method="estimate",tumor = T,indications = brca_vector)

write.table(res_estimate,"estimate.txt",sep="\t",row.names = F)

res_estimate$cell_type=paste0(res_estimate$cell_type,"_ESTIMATE")

#TIMER

exp=exprMatrix

res_timer <- deconvolute(exp, method="timer",tumor = T,indications = brca_vector)

write.table(res_timer,"timer.txt",sep="\t",row.names = F)

res_timer$cell_type=paste0(res_timer$cell_type,"_TIMER")

#ABIS

exp=exprMatrix

res_abis <- deconvolute(exp, method="abis",tumor = T,indications = brca_vector)

write.table(res_abis,"abis.txt",sep="\t",row.names = F)

res_abis$cell_type=paste0(res_abis$cell_type,"_ABIS")

#ConsensusTME

exp=exprMatrix

res_consensus_tme <- deconvolute(exp, method="consensus_tme",tumor = T,indications = brca_vector)

write.table(res_consensus_tme,"consensus_tme.txt",sep="\t",row.names = F)

res_consensus_tme$cell_type=paste0(res_consensus_tme$cell_type,"_ConsensusTME")

#xCell

exp=exprMatrix

res_xcell <- deconvolute(exp, method="xcell",tumor = T,indications = brca_vector)

write.table(res_xcell,"xcell.txt",sep="\t",row.names = F)

res_xcell$cell_type=paste0(res_xcell$cell_type,"_xCell")

#EPIC

exp=exprMatrix

res_epic <- deconvolute(exp, method="epic",tumor = T,indications = brca_vector)

write.table(res_epic,"epic.txt",sep="\t",row.names = F)

res_epic$cell_type=paste0(res_epic$cell_type,"_EPIC")

#quanTIseq

exp=exprMatrix

res_quantiseq <- deconvolute(exp, method="quantiseq",tumor = T,indications = brca_vector)

write.table(res_quantiseq,"quantiseq.txt",sep="\t",row.names = F)

res_quantiseq$cell_type=paste0(res_quantiseq$cell_type,"_quanTIseq")

#CIBERSORT

data=exprMatrix

v=voom(data, plot=F, save.plot=F)

out=v$E

out=rbind(ID=colnames(out), out)

write.table(out,file="uniq.symbol.txt",sep="\t",quote=F,col.names=F)

source("CIBERSORT.R",encoding = "utf-8")

results=CIBERSORT("LM22.txt", "uniq.symbol.txt", perm=1000, QN=TRUE)

res_CIBERSORT=read.table("CIBERSORT-Results.txt",sep="\t",header=T,row.names=1,check.names=F)

res_CIBERSORT=t(res_CIBERSORT)

rownames(res_CIBERSORT)=paste0(rownames(res_CIBERSORT),"_CIBERSORT")

res_CIBERSORT=data.frame(cell_type=rownames(res_CIBERSORT),res_CIBERSORT)

res_consensus_tme=res_consensus_tme[1:(nrow(res_consensus_tme)-1),]

res_xcell=res_xcell[1:(nrow(res_xcell)-3),]

res_CIBERSORT=res_CIBERSORT[1:(nrow(res_CIBERSORT)-3),]

res=rbind(res_abis,res_consensus_tme,res_epic,res_quantiseq,res_timer,res_xcell,res_CIBERSORT)

res=as.matrix(res)

rownames(res)=res[,1]

exp=res[,2:ncol(res)]

dimnames=list(rownames(exp),colnames(exp))

data=matrix(as.numeric(as.matrix(exp)),nrow=nrow(exp),dimnames=dimnames)

gene="FCRL5"

gene_exp=exprMatrix[gene,]

gene_exp=t(gene_exp)

samesample=intersect(rownames(gene_exp),colnames(data))

gene_exp=gene_exp[samesample,]

gene_exp=as.data.frame(gene_exp)

colnames(gene_exp)=gene

data=data[,samesample]

x=as.numeric(gene_exp[,1])

outTab=data.frame()

for(i in rownames(data)){

y=as.numeric(data[i,])

if(sd(y)<0.001){next}

corT=cor.test(x, y, method="spearman")

cor=corT$estimate

pvalue=corT$p.value

if(pvalue<0.05){

outTab=rbind(outTab,cbind(immune=i, cor, pvalue))

}

}

write.table(file="corResult.txt", outTab, sep="\t", quote=F, row.names=F)

corResult=read.table("corResult.txt", head=T, sep="\t")

corResult$Software=sapply(strsplit(corResult[,1],"_"), '[', 2)

corResult$Software=factor(corResult$Software,level=as.character(unique(corResult$Software[rev(order(as.character(corResult$Software)))])))

b=corResult[order(corResult$Software),]

b$immune=factor(b$immune,levels=rev(as.character(b$immune)))

colslabels=rep(hue_pal()(length(levels(b$Software))),table(b$Software))

pdf(file="correlation.pdf", width=9, height=10)

ggplot(data=b, aes(x=cor, y=immune, color=Software))+

labs(x="Correlation coefficient",y="Immune cell")+

geom_point(size=4.1)+

theme(panel.background=element_rect(fill="white",size=1,color="black"),

panel.grid=element_line(color="grey75",size=0.5),

axis.ticks = element_line(size=0.5),

axis.text.y = ggtext::element_markdown(colour=rev(colslabels)))

dev.off()

sessionInfo()
